# Dynamic Atomistic Polar Structure Underpins Ultrahigh Linear Electro-Optic Coefficient in Transparent Ferroelectric Ceramics

**DOI:** 10.1021/jacs.5c15699

**Published:** 2025-11-04

**Authors:** Qinghui Jiang, Weigang Zhao, Man Zhang, Jian-Ping Zhou, Mingqing Liao, Andriy Smolyanyuk, Zixuan Wu, Chenglong Jia, Xiaoyong Wei, Cedric Weber, Nadezda V. Tarakina, Isaac Abrahams, Jan M. Tomczak, Zi-Kui Liu, Vladimir Roddatis, Haixue Yan

**Affiliations:** † State Key Laboratory of Materials Processing and Die and Mould Technology, and School of Materials Science and Engineering, 12443 Huazhong University of Science and Technology, Wuhan 430074, P. R. China; ‡ Electronic Materials Research Laboratory, Key Laboratory of the Ministry of Education & International Center for Dielectric Research, School of Electronic Science and Engineering, 12480 Xi’an Jiaotong University, Xi’an 710049, P. R. China; § School of Mechanical Engineering, 4468 University of Leeds, Leeds LS2 9JT, U.K.; ∥ School of Engineering and Materials Science, 4617 Queen Mary University of London, London E1 4NS, U.K.; ⊥ School of Physics and Information Technology, 530448 Shaanxi Normal University, Xi’an 710119, P. R. China; # School of Materials Science and Engineering, 12676 Jiangsu University of Science and Technology, Zhenjiang 212003, P. R. China; ∇ Institute of Solid State Physics, 27259 TU Wien, Vienna 1040, Austria; ○ School of Physical Science and Technology, 12426 Lanzhou University, Lanzhou 730000, P. R. China; ◆ Quantum Brilliance Pty, The Australian National University, Canberra, Australian Capital Territory 2600, Australia; ¶ 28321 Max Planck Institute of Colloids and Interfaces, Department of Colloid Chemistry, Potsdam 14476, Germany; †† Department of Chemistry, Queen Mary University of London, London E1 4NS, U.K.; ‡‡ Department of Physics, 4616 King’s College London, London WC2R 2LS, U.K.; §§ Department of Materials Science and Engineering, College of Earth and Mineral Science, The Pennsylvania State University, University Park, Pennsylvania 16802, United States; ∥∥ 28337 GFZ Helmholtz Centre for Geosciences, Telegrafenberg, Potsdam D-14473, Germany

## Abstract

Transparent ferroelectrics with high linear electro-optic (EO) coefficients are critical for advanced electro-optical devices. However, achieving optical transparency in ferroelectric ceramics remains challenging due to visible light scattering caused by defects such as domain walls, grain boundaries, and pores. Here, we report the successful fabrication of transparent ferroelectric ceramics through innovative chemical composition design and an advanced two-step sintering process in the La-doped Pb­(Mg_1/3_Nb_2/3_)­O_3_–PbTiO_3_ system. The optical transparency, which is near the theoretical upper limit, can be attributed to the wide band gap and the minimization of light scattering of defects. By minimizing porosity and engineering grain/domain sizes to differ significantly from the wavelengths of visible light, we suppress scattering, achieving optical transparency near the theoretical upper limit. Strikingly, these ceramics exhibit an ultrahigh linear EO coefficient of ∼1417 pm/V, over 65 times greater than that of LiNbO_3_ single crystals, the current industry standard. We attribute this exceptional performance to dynamic atomistic polar structures within switchable, thermally stable domains, which enhance electronic polarization sensitivity. This mechanism is corroborated by dielectric spectroscopy, high-resolution transmission electron microscopy and simulation. Our findings offer insights into the design of cost-effective transparent materials with exceptional EO properties, paving the way for next-generation electro-optical devices.

## Graphical abstract

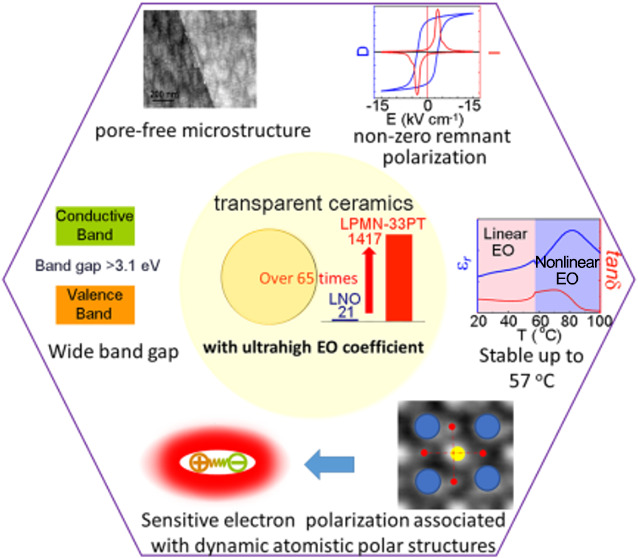

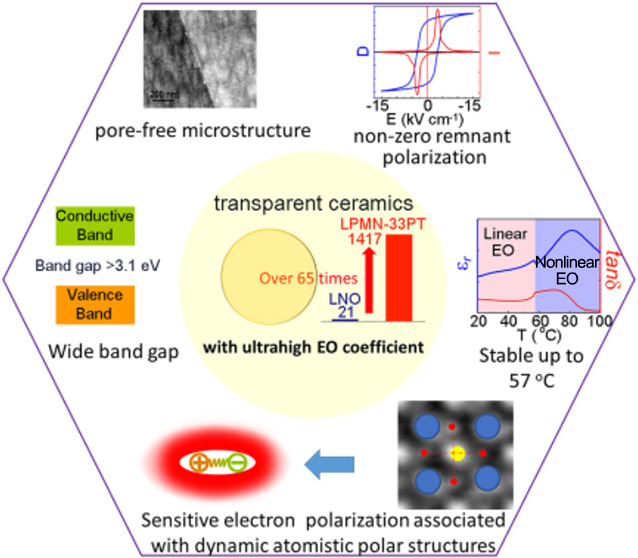

## 1 Introduction

Materials that exhibit a high linear electro-optic (EO) coefficient are utilized in a variety of applications including sensing, robotics and optical modulation. Such linear EO properties can occur in transparent noncentrosymmetric structured materials and are related to changes in dielectric permittivity under applied voltage. Ferroelectrics are a group of noncentrosymmetric structured materials which are characterized by switchable spontaneous polarization. Ferroelectric single crystal lithium niobate (LiNbO_3_) has been widely used for EO devices, but its low EO coefficient (γ_c_ ∼21 pm/V) means that large crystals with longer path lengths are required, limiting miniaturization, while the high drive voltage needed to change the permittivity adds a significant cost to the power supply.^1,2^ For linear EO applications, materials must be transparent in the visible light frequency range. However, a key challenge for the fabrication of transparent ferroelectric single crystals is the presence of domain walls, which act as scattering sources that limit transparency.

Domain engineering by ac poling has recently been reported to be effective in the preparation of transparent ferroelectric single crystals with ultrahigh piezoelectric coefficients.^3^ Similarly, transparent ferroelectric single crystals with high linear EO coefficient values have been prepared using high temperature poling.^1^ However, the growth of ferroelectric single crystals is an expensive process, and the properties of the resulting single crystals can vary due to continuous changes in composition along the direction of crystal growth.^4,5^

The development of highly transparent EO ceramics would have advantages over single crystals in terms of cost and composition/property control. To achieve this in ceramics, it is necessary to minimize the light scattering effects associated with porosity,^6,7^ grain boundaries and domain walls. Additionally, the developed ferroelectrics must have a band gap greater than 3.1 eV, associated with the shortest wavelength of visible light at the ultraviolet color edge, and should exhibit a sensitive voltage induced structural change, to facilitate a high linear EO effect.

It is possible to develop highly transparent ceramics in the prototypical (100-*x*)­Pb­(Mg_1/3_Nb_2/3_)­O_3_-*x*PbTiO_3_ (PMN-*x*PT) relaxor ferroelectrics with *x* < 40 because their domain sizes are much smaller than the wavelength range of visible light.^8,9^ Compositions in the PMN-*x*PT system near the morphotropic phase boundary (MPB) have the potential to exhibit strong linear EO properties as a result of their nano polar structures. Additionally, in related systems, substitution of Pb^2+^ by La^3+^ is known to increase optical transparency.^10,11^ In the present study, we combine chemical composition and nanopolar structure design, with a novel processing method, to prepare transparent ceramics with ultrahigh linear EO coefficients. Supported by experimental and simulation work, a dynamic atomistic polar structure model is proposed to explain this phenomenon.

## 2 Experimental Section

### 2.1 Ceramics Fabrication

Powders of composition La_2.5_Pb_96.25_Mg_(100–*x*)/3_Nb_2(100–*x*)/3_Ti_*x*_O_300_ (LPMN-*x*PT) were synthesized by the columbite precursor method which can inhibit the generation of the pyrochlore phase that seriously affects the ferroelectric and dielectric properties of ceramics. High purity MgO (99.90%), Nb_2_O_5_ (99.99%), TiO_2_ (99.99%), La_2_O_3_ (99.99%), and PbO (99.90%) were used as raw materials. MgO and Nb_2_O_5_ powders were homogeneously mixed and ground in an agate mortar for 30 min. Following this, the mixed powders were transferred to an Al_2_O_3_ crucible and calcined at 1100 °C for 10 h. After cooling to ambient temperature, the powders were crushed, ground for another 30 min, and calcined at 1200 °C for 4 h to produce single-phase MgNb_2_O_6_. Subsequently, MgNb_2_O_6_ was combined with TiO_2_, La_2_O_3_, and PbO (4 mol % excess) in an agate mortar, manually ground for 30 min and calcined in an Al_2_O_3_ crucible at two temperatures: 800 and 900 °C, each for a duration of 2 h. This process yielded single-phase LPMN-*x*PT powders. The powders, which were high-energy ball milled for 5 h with an SPEX 8000 M mill, were then loaded into a graphite die with a diameter of 15 ∼ 20 mm and sintered at 950 °C for 5–20 min under a pressure of 50 MPa using spark plasma sintering (SPS, HPD 25/1, FCT Systeme GmbH, Germany). Finally, the formed pellets underwent a second sintering process between 1150 and 1250 °C for 6 h under flowing oxygen. The oxygen flow rate was 20 mL/min with a gas pressure of 0.13 MPa.

### 2.2 SEM Measurement and Density

LPMN-*x*PT ceramics were manually ground using abrasive papers of varying roughness. They were then polished with a nanoalumina suspension and thermally etched at 900 °C for 15 min to delineate the grain boundaries. The microstructural morphology was observed using a Nova NanoSEM 450. The Archimedes method was used to check the density of samples through the displacement of water (∼99.9% relative density).

### 2.3 XRD Analysis

X-ray powder diffraction measurements were performed on a PANalytical Cubix3 diffractometer fitted with a PIXcel (1D) detector, using Ni filtered Cu–Kα radiation (λ = 1.5418 Å). Data were collected at room temperature in flat plate Bragg–Brentano geometry over the 2θ range of 5–120° in steps of 0.0315° for an effective count time of 200 s per step. Structure refinement for the *x* = 33 composition was carried out by Rietveld analysis using the GSAS suite of programs.^12^ Initial starting models were based on the structures reported by Araujo et al.^13^ in space group *P*4*mm*, and Singh and Pandy^14^ in space group *Pm*. Other models in space groups *Cm* and *R*3*m* were also tested. For the unpoled sample a significantly better fit (as determined by the method of Hamilton^15^) was obtained using a two-phase model consisting of *P*4*mm* and *Pm* phases, while for poled samples (at fields of 1.8 and 10 kV/cm) a single-phase model consisting only of the *Pm* phase was found to give the best fit.

### 2.4 TEM Measurements

One set of TEM specimens was conventionally prepared by mechanical polishing followed by Ar^+^ ion milling using a Gatan PIPS-II operated at acceleration voltages between 2 and 4 kV. TEM specimens were also prepared by the lift-out method using a Thermo Fisher Scientific (TFS, formerly FEI) Helios G4 UC dual beam instrument. The final polishing of thin TEM lamellae was done using a Gatan PIPS-II device with Ar^+^-ions accelerated with 0.1 V. A TFS Themis Z 80–300 microscope was used to collect High Angle Annular Dark-Field (HAADF) and integrated Differential Phase Contrast (iDPC) images as well as Selected-Area Electron Diffraction (SAED) patterns. The microscope was equipped with a Cs-corrector at the probe side, a SuperX Energy Dispersive X-ray spectrometer, and a Gatan Imaging Filter (GIF) Continuum 1065ER. The imaging was carried out with a convergence/collection semiangle of 30 mrad/26–155 mrad for HAADF and 30 mrad/6–24 mrad for the DF4 (iDPC) detector, respectively. All measured LPMN-PT (*x* = 29, 33, 36) ceramics were nonconductive, and consequently the corresponding TEM specimens demonstrated the usual effects (drift, instabilities, etc.) of charging while illuminated by electrons. To avoid these undesirable effects, the specimens were coated with a thin (3–6 nm) carbon film and moderate beam currents of 10–40 pA were used. Nevertheless, images taken from several areas were required to illustrate features of the LPMN-*x*PT sample structure and changes stimulated by the electron beam.

### 2.5 Optical Transmittance Measurements

Transmission spectra were measured with a UV–visible near-infrared spectrophotometer (SolidSpec-3700, Shimadzu) at wavelengths ranging from 300 to 1600 nm using an integrating sphere. According to the Fresnel equations, the reflection loss at two faces and transmittance of the ceramic plates were respectively calculated through $R=\frac{{\left(n-1\right)}^{2}}{n^{2}+1}$ and $T=\frac{2n}{n^{2}+1}$ , where *n* is the wavelength-dependent refractive index. *n* values of PMN-*x*PT single crystals^16^ were used to calculate *R* and *T* values. In single crystal PMN-33PT, an *n* value of 2.6 at 633 nm yields an *R* value of 33% and a *T* value of 67%.

### 2.6 Dielectric, Piezoelectric and Ferroelectric Measurements

Ag electrode paste was applied to both sides of polished ceramic pellets for electrical property measurements and pellets fired at 600 °C for 15 min prior to measurements. The temperature dependencies of the dielectric permittivity and loss at different frequencies of the samples were measured using an LCR meter (Agilent Technologies Ltd., 4284A, Kobe, Hyogo, Japan) connected to a furnace. The dielectric displacement-electric field (*D*–*E*) and current-electric field (*I*–*E*) hysteresis loops of LPMN-*x*PT ceramics were measured using a ferroelectric hysteresis measurement tester (NPL, U.K.). The electrostrictive curves of LPMN-*x*PT ceramics were measured using a ferroelectric integrated test system (airACCT-2000E, Germany). Samples for piezoelectric measurements were poled at room temperature under various dc electric fields. The piezoelectric coefficient, *d*_33_, of poled samples was measured using a quasi-static *d*_33_ meter (CAS, ZJ-3B).

### 2.7 Electro-Optic Measurements

The effective electro-optic coefficient (EOC, γ_c_) was measured by the minimum-transmission point measurement method with a 633 nm He–Ne laser as the light source. The presence of an EO effect in the sample retards the phase of polarized light with $\varphi_{E}=\frac{2\pi }{\lambda }\Delta \mathrm{nl}$ . The sample size was 4 mm × 6 mm with a thickness of 0.16 mm. Measurement details and reliability evaluation are summarized in Figures S1 and S2. Measurements were carried out in triplicate.

### 2.8 Simulations

Electronic structure calculations were performed starting from the experimentally determined space-groups and atomic positions. Chemical disorder was accounted for by generating all minimal unit-cells compatible with the approximated composition Pb­(Ti_*x*_Nb_*y*_Mg_*z*_)­O_3_ with *x* = 3/9, *y* = 4/9, *z* = 2/9 using enumlib.^17–19^ The resulting 10,036 [3,288] unit-cells for structures with parent space group *Pm* (#6) [*P*4*mm* (#99)] were first curated according to their total energies, estimated from density functional theory using VASP^20–23^ for a single k-point, the PBE exchange correlation potential, a plane-wave cutoff of 600 eV, and a Gaussian smearing of 0.04 eV. Reranking the 10 configurations with the lowest total energy using a full Brillouin sampling, singled out four low-energy configurations differing by less than 2 meV/atom in total energy, while the next highest energy configuration was more than 15 meV/atom away. The three most stable configurations with parent space-group *Pm* were then internally relaxed. Using Wien2k^24,25^ with 32 reducible k-points, a basis set cutoff RK_max_ = 7, and the LDA potential, the configurations were again reranked according to their total energy. The energetic differences between poled and unpoled structures for a given configuration (∼O (0.04 mRy/atom)) were of the same order as differences between configurations (∼O (0.03 mRy/atom)). The poling-induced spontaneous electrical polarization was then determined for a given configuration by subtracting results for the unpoled from the poled structure using BerryPI.^26^ The two most stable configurations yielded polarizations comparable to BaTiO_3_ (0.3 C/m^2^), while the next, less stable, configuration had a polarization smaller by 1 order of magnitude. The multiscale entropy approach (recently termed as zentropy theory^27,28^ ) was used to predict the free energy of the Pb­(Ti_1/3_Nb_4/9_Mg_2/9_)­O_3_ system. In the zentropy theory, the total entropy is expressed as *S* = −*k*_B_∑_*k*_ *p* *_k_* ln*p* *_k_* + ∑_*k*_ *p* *_k_* *S* *_k_* where the subscript represents a configuration with an entropy of *S* *_k_* that the system experiences with the probability $p_{k}=\frac{e^{-F_{k}/k_{B}T}}{\underset{i}{\sum }e^{-F_{i}/k_{B}T}}$ and *F* *_k_* being the free energy of configuration *k* and the summation over all configurations. The free energy of the system is then *F* = ∑_*k*_ *p* *_k_* *F* *_k_* + *k*_*B*_ *T*∑_*k*_ *p* *_k_* ln*p* *_k_*. The atomic configuration with the lowest energy from the above exploration is regarded as the ground-state configuration, and the exploration of other nonground-state configurations is discussed in the Supporting text along with the *ab initio* calculations of their energies.

## 3 Results and Discussion

### 3.1 Design and Preparation of Transparent Ceramics with High Linear EO Coefficient

We used an advanced two-step method to sinter La doped PMN-*x*PT (LPMN-*x*PT) EO ceramics, the first step of which involved spark plasma sintering (SPS),^29,30^ resulting in ceramics with high relative density and minimal porosity. In the second step, ceramics were sintered again under oxygen-rich conditions, to increase the grain size to further minimize the scattering effects from grain boundaries. The inset of Figure 1A shows a photograph of transparent LPMN-*x*PT ceramics, confirming their high optical transparency. Transparency in ceramics requires them to have high relative density with grain sizes much larger than the wavelength of red light (ca. 0.7 μm). Indeed, all the studied ceramics had relative densities of around 99.9%. There are no observed micro pores within or between grains. The grain size is about 10 ∼ 20 μm (Figure S3). Notably, the large grain size means that the density of grain boundaries is low, which helps to minimize light scattering as needed for high transparency.^31^ As shown in Figure 1A, all the ceramics exhibit percentage transparency values of around 67% in the visible range, which represents the theoretical maximum based on their refractive indices (see Table S1).^3^ Low transparency is seen for all the studied ceramics below ca. 400 nm corresponding to the band gap energy (ca. 3.1 eV).

**Figure 1:**
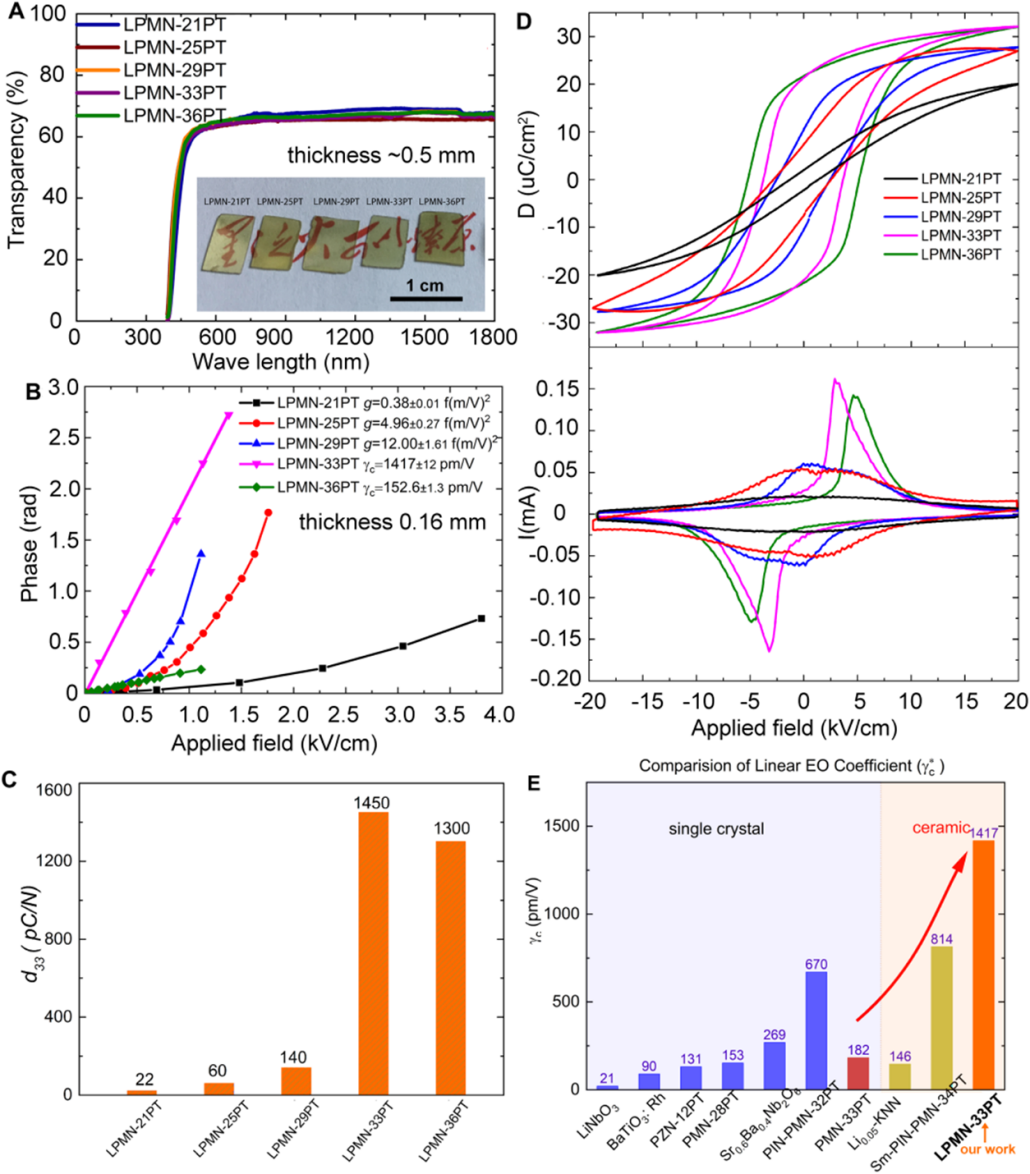
Electro-optic properties of transparent LPMN-*x*PT ceramics. (A) Wavelength dependence of transparency (inset: photograph of transparent LPMN-*x*PT ceramics showing written text underneath); (B) optical phase shift (φ_*E*_) under applied field (the samples were poled at 5 kV/cm for 10 min before EO measurement); (C) effect of composition on the piezoelectric coefficient, *d*_33_ (the *d*_33_ value was measured after a ferroelectric polarization hysteresis test which served as ac poling); (D) ferroelectric properties measured at 1 Hz and room temperature showing displacement-electric field (upper) and current-electric field (lower) loops; (E) linear electro-optic coefficient, γ_c_, values for ferroelectric single crystals and ceramics with crystallographic direction indicated (LiNbO_3_,^37^ BaTiO_3_,^38^ 0.88Pb­(Zn_1/3_Nb_2/3_)­O_3_–0.12PbTiO_3_ (PNZ-12PT),^39^ Sr_0.6_Ba_0.4_Nb_2_O_6_,^40^ 0.21Pb­(In_1/2_Nb_1/2_)­O_3_–0.47Pb­(Mg_1/3_Nb_2/3_)­O_3_–0.32PbTiO_3_ (PIN–PMN–32PT),^1^ PMN-28PT,^3^ and PMN-33PT^16^ single crystals, Li_0.05_[(K_0.5_Na_0.5_)_0.95_]­NbO_3_ ^41^ and 1.0 mol % Sm-doped 24PIN-42PMN-34PT^42^ ceramics).

Prior to poling, ferroelectric ceramics do not show linear EO properties as their local polar structures are randomly oriented leading to average centrosymmetric structures. After poling, domain switching and irreversible field induced phase transitions lead to long-range ordering of dipoles and hence a noncentrosymmetric structure. The structure of poled ceramics is thermally stable provided the depoling temperature is above ambient temperature. In the case of PMN-*x*PT based ceramics, the depoling temperature is associated with the phase transition between monoclinic and tetragonal phases.^32,33^ Figure 1B shows the optical phase shift under applied field for the studied LPMN-*x*PT ceramics. Both LPMN-33PT and LPMN-36PT ceramics show linear EO behavior, but the compositions with lower PT concentrations show nonlinear EO behavior under high applied field, which indicates that the depoling temperatures of the *x* = 33 and 36 compositions are above room temperature, while those for the other compositions are near or below room temperature. While it is challenging to prepare linear-EO ceramics, for applications, they are preferable to nonlinear EO systems because they show greater sensitivity at lower voltages. The higher thermal depoling temperatures of LPMN-33PT and LPMN-36PT ceramics are consistent with their high temperatures of maximum permittivity, *T*_m_ (Figure S4). The different thermal stabilities of the LPMN-*x*PT ceramics are also consistent with their piezoelectric coefficient (*d*_33_) values (Figure 1C). The very low *d*_33_ values for compositions with *x* ≤ 29 suggest that their noncentrosymmetric structures induced by poling are thermally unstable at room temperature, associated with significant aging (ca. 50% decrease in the value of *d*_33_ over 24 h). In other words, the field induced transition from weak polar to strong polar in the low *x*-value compositions is mainly reversible, which is supported by the low remnant polarization seen in the dielectric displacement-electric field (*D*–*E*) loops and four broad current peaks, one in each quadrant, in the current-electric field (*I*–*E*) loops (Figure 1D).^34,35^ In contrast, the poled LPMN-33PT and LPMN-36PT ceramics show linear EO behavior, indicating that the field induced domain switching and phase transition in these ceramics are irreversible, consistent with their high remnant polarization associated with two sharp current peaks, one in the first quadrant and one in the third, of their *I*–*E* loops (Figure 1D).^36^ The field induced polarization of LPMN-25PT is very close to that of LPMN-29PT, which can be attributed to the contribution of field induced conductivity in LPMN-25PT. For these high *x*-value compositions, the poled structure is thermally stable, leading to a nonzero *d*_33_ value after poling, with no significant change in this value over several weeks. Indeed, the piezoelectric coefficient of LPMN-33PT is 1450 pC/N after dc poling (Figure 1C and Table S2). It should be noted here that the *d*_33_ value of the LPMN-33PT ceramic after ac poling was lower than that after dc poling, indicating that the ceramic was not fully poled during the ac poling. Rietveld analysis of X-ray powder diffraction (XRD) data on the LPMN-33PT ceramic reveals a mixture of monoclinic (space group *Pm*) and tetragonal (space group *P*4*mm*) phases in a wt % ratio of 1:2, with conversion to the monoclinic phase after poling at fields in the range 1.8–10 kV/cm (Figure S5 and Table S3 and S4).

A comparison of the linear EO coefficient (γ_c_) for the LPMN-33PT ceramic with those of the best performing bulk materials reported to date is shown in Figure 1E. It is worth noting that both LPMN-33PT and LPMN-36PT show high piezoelectric coefficients in Figure 1C, but only LPMN-33PT shows an ultrahigh linear EO coefficient in Figure 1E. The field induced strain of LPMN-36PT is higher than that of LPMN-33PT (Figure S6). There is a contribution from the converse piezoelectric coefficient, *d*_31_, to the measured linear EO coefficient. Generally, in ceramics the value of *d*_31_ is roughly half of that of the converse *d*_33_ coefficient, which is related to the slope of the strain-electric field curve in Figure S6a. The slope of LPMN-33PT is slightly lower than that of LPMN-36PT in Figure S6a, which indicates that the converse *d*_31_ coefficient of LPMN-33PT is lower than that of LPMN-36PT. Given also that the EO coefficient of LPMN-36PT is much lower than that of LPMN-33PT, then the measured linear EO coefficient of LPMN-33PT likely only has a minor contribution from the converse piezoelectric effect, which indicates that the major contribution to the linear EO coefficient can be attributed to the change of permittivity under applied field. The LPMN-33PT ceramic exhibits the highest linear EO coefficient, for a bulk material, reported to date, more than double that of the best performing single crystal. Indeed, our measurement setup (Figure S1) was calibrated using commercially available LiNbO_3_ single crystals (Figure S2). The high EO coefficient of LPMN-33PT compared to single crystals highlights the advantages of ceramics in terms of optimizing domain structure and compositional homogeneity.

The linear EO coefficient is related to changes of dielectric permittivity at visible light frequencies under applied voltage^1,43^ and in theory, only the contribution of electronic polarization to dielectric permittivity is active at these frequencies. In the literature,^1,44^ there is clear experimental evidence to show that perovskite ferroelectrics with high domain wall density have very good linear EO properties, but the exact physical mechanisms are still unclear.

### 3.2 Thermal Stability of Electro-Optic, Dielectric and Ferroelectric Properties

Thermal stability measurements show that the linear EO coefficient of LPMN-33PT remains thermally stable up to 57 °C (Figure 2A). Above this temperature, the sample shows only nonlinear EO behavior. A corresponding frequency independent peak at 57 °C in the relative permittivity and loss profiles (Figure 2B) is consistent with a transition associated with a depoling of the poled ceramic structure. Indeed, a phase transition from monoclinic and tetragonal polar phases on heating has been reported in the PMN–PT system at 60 °C,^32,33,45^ with the slight lowering of the transition temperature observed in the present case attributable to the La doping,^11^ which indicates that the depoling temperature at 57 °C is associated with the monoclinic-tetragonal phase transition. Also evident in Figure 2B, is a broad frequency dependent peak at around 80 °C, which is typical of relaxor ferroelectric behavior.^46^ Furthermore, as shown in Figure 2B, below 50 °C, the dielectric permittivity increases and the dielectric loss decreases with increasing temperature. The increase in dielectric permittivity is related to the enhanced dipole response under the application of the ac field, while the decrease in loss is attributed to the reduction in interdipole friction at high temperatures.

**Figure 2:**
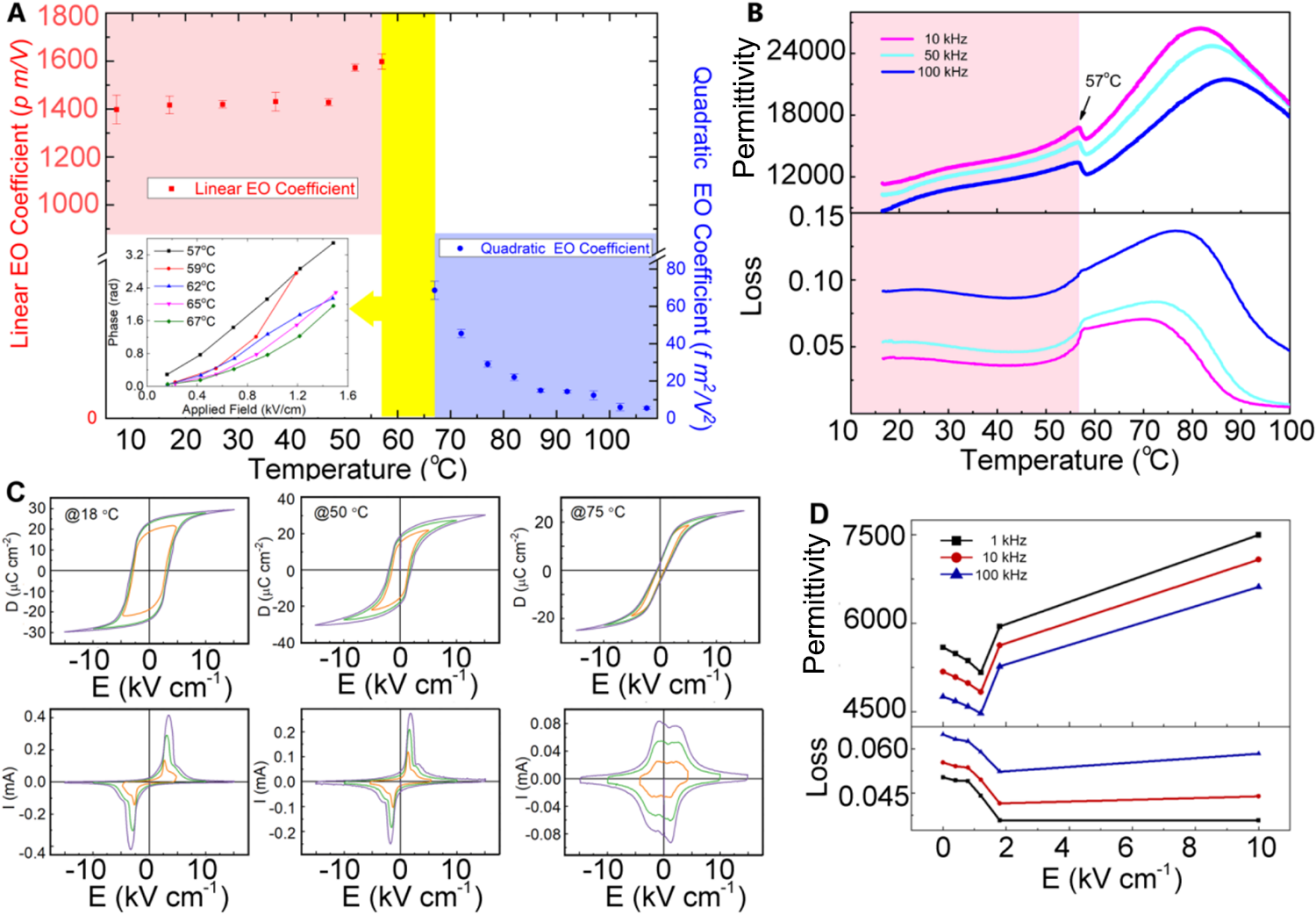
Temperature dependencies of electro-optic, dielectric and ferroelectric properties of poled LPMN-33PT ceramics. (A) Electro-optic coefficient with optical phase shift under applied field at selected temperatures inset; (B) relative permittivity and loss; (C) ferroelectric properties at different temperatures [displacement-electric field (*D*–*E*) (upper) and current-electric field (*I*–*E*) (lower) loops are shown]; (D) effect of poling field on relative permittivity and loss.

Below 57 °C, the long-range ordered polar structure consists of thermally stable domains which have preferred orientation caused by poling and is associated with a high remnant polarization of around 20 μC/cm^2^ (Figure 2C) and current peaks in the first and third quadrants of the corresponding *I*–*E* loops. The presence of a high-field shoulder on the current peaks indicates two processes occur on poling, viz.: domain switching and an irreversible field induced phase transition.^36^ This proposition is supported by the observation of an initial decrease and subsequent increase in relative permittivity immediately after poling (Figure 2D).^47,48^ The results are consistent with the XRD data (Figure S5 and Tables S3 and S4), which confirm a transition from a mixture of monoclinic and tetragonal polar phases to the pure monoclinic phase after poling. The *D*–*E* loops measured at 75 °C show very low remnant polarization with nominally single broad current peaks in each of the four quadrants in the corresponding *I*–*E* loops, indicative of a reversible field-induced phase transition.^34,35^ The results suggest, that above 57 °C, depoling occurs, which would be expected to be associated with a transition from noncentrosymmetric to average centrosymmetric symmetry.

The phenomenon of the ultrahigh linear EO coefficient Figures 1E and 2A cannot be explained simply by the presence of a thermally stable domain structure below 57 °C. The dielectric relaxation frequency of domain walls, including boundaries between polar nanodomains, is much lower than the visible light frequency range and hence domain walls, as a group, are not active at these frequencies but local atomistic dipoles within domain walls are active even at THz frequencies.^49^ It is reasonable to expect, that changes in the electronic polarization under applied voltage, associated with local atomistic dipoles with reversible rotation, are responsible for the high EO coefficient. We propose that there is a high density of dynamic atomistic level dipoles (ergodic in nature), dispersed in the switchable and thermally stable long-range ordered submicron domains. This results in highly sensitive electronic polarization leading to a high linear EO coefficient in the ceramic, with a nonzero *d*_33_ value after poling. Our proposed dynamic atomistic polar structure model is supported by high-resolution TEM experimental work and simulation work with details below.

### 3.3 TEM Results of Transparent Ceramics

Figure S7a shows low-magnification scanning transmission electron microscopy (STEM) images of the *x* = 29, 33, and 36 ceramics. There are no visible domains in the image for the *x* = 29 sample. In contrast, the images for the higher *x*-value compositions show the presence of lamellar-shaped domains of various sizes. Similar domain structures were also recently observed in transmission electron microscopy (TEM) images of PMN-*x*PT ceramics.^32^ In all the studied LPMN-*x*PT compositions, selected area electron diffraction (SAED) patterns collected from individual grains along low index crystallographic directions show no additional spots (Figure S7b). Moreover, no variation of chemical composition or elemental segregation was revealed by Energy Dispersive X-ray (EDX) spectroscopy in poled and unpoled samples (Figure S8).

In thin parts of TEM specimens, microdomain contrast is lost, which suggests there are small sized local polar structures within a microdomain. The microdomains become unstable in thin areas of a TEM sample because there are not enough local polar structures to build up stable correlations. High angle annular dark field scanning transmission electron microscopy (HAADF) and integrated differential phase contrast (iDPC) images collected along [100] are shown in Figure 3 and were used to measure the shifts of B-cations at atomic column resolution. In previous studies of related materials, polar nanodomains were typically observed in very thin areas,^50–53^ while with increasing ceramic thickness, lamella-like and bulk^1^ domains are visible. However, in the present case, no evidence for the presence of polar nanodomains was found in the B-cation displacement map (Figure 3B), possibly due to the thickness of the sample (Figure S9). Moreover, the average shift of B-cations is only ca. 3.3 pm, which is much smaller than the experimental pixel size. In contrast, random displacements of oxygen atomic columns are visible in the iDPC image (Figure 3C). It is proposed here that these oxygen atom shifts are the root cause of the easy susceptibility to external mechanical and electrical stimuli. The local structures associated with the oxygen atomic columns are atomistic scale polar structures. This is also consistent with recent findings that there are high density low-angle nanodomain walls in PMN–PT, facilitated by the nanoscale polar character and lattice strain disorder.^8,32^ The atomistic scale polar structures are extremely small in size and locally dynamic. The electronic polarization associated with the local dynamic atomistic scale polar structures could be the origin for the observed ultrahigh linear EO coefficient in the present system.

**Figure 3:**
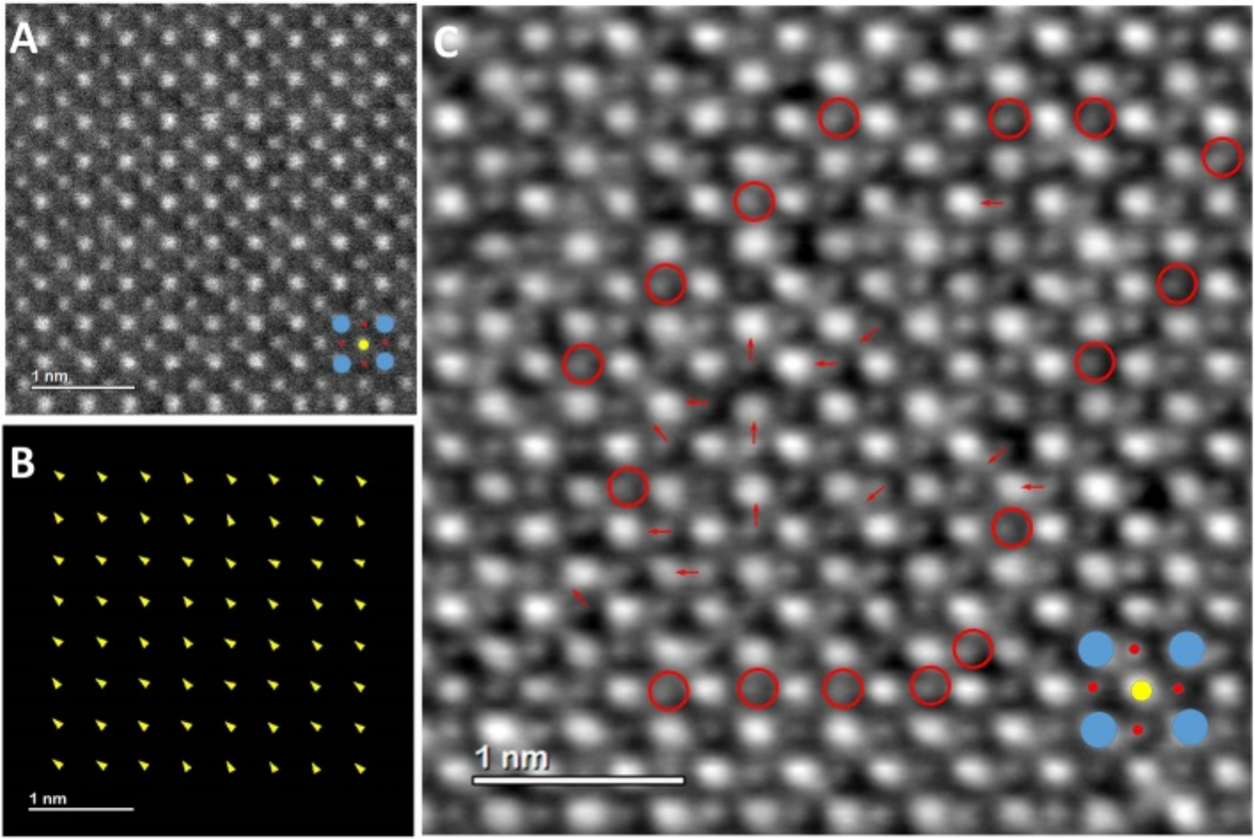
HAADF image (A) corresponding B-cation displacement map (B) and iDPC image (C) of LPMN-33PT ceramic. The pixel size of experimental HAADF and iDPC images is 9.168 pm. The average length of the displacement vector is about 3.3 pm. Simplified atomic models showing positions of A- (blue) and B- cations (yellow) as well as oxygen atoms (red) are indicated. In the iDPC image, positions of oxygen atomic columns visible in the center of squares formed by A- and B- cation unit cells are marked with red circles, while arbitrary displaced O-columns are shown with red arrowheads.

### 3.4 Simulation Work

Our proposed mechanism is supported by first-principles electronic structure simulations. Starting from the experimental crystal structures of LPMN-33PT prior to and after poling, we assessed the stability of the field-induced structural changes, allowing for, both chemical disorder and relaxation effects. For the two most stable disorder configurations (see Section 2.8 and Table S5 and Figure S10), we find that poling induces a polarization difference in the order 20 μC/cm^2^. This sizable polarization indicates that the poling field induces structure changes and suggests that the poled structures are structurally metastable.

The ultrahigh linear EO coefficient of LPMN-33PT can be further explained using the recently developed zentropy theory^54^ that considers a phase at finite temperatures as a statistical mixture of a set of configurations that the system experiences in terms of the concept of statistical mechanics developed by Gibbs.^55^ In the zentropy theory, one starts from the ground-state configuration of a system at 0 K as shown in Table S5. By exhausting all internal degrees of freedom, symmetry-breaking nonground-state configurations are explored with their free energies predicted from DFT-based *ab initio* calculations.^56^

Based on the zentropy theory, the PMN-33PT system at finite temperatures is a mixture of the no domain wall (NoDW) ground-state configuration and nonground-state configurations. In PbTiO_3_, the polarization is the same for all Ti atoms, so the only internal degrees of freedom are the switching of polarization directions for the formation of 90- and 180-degree domain walls. In PMN-33PT, there are other internal degrees of freedom due to the different polarization directions of the different B-site atoms (Mg, Nb, and Ti) in the NoDW ground-state configuration as shown in Figure S11, in addition to the four types of domain walls in orthorhombic-like PMN-33PT, i.e., 60°, 90°, 120°, and 180° (DW60/90/120/180).^57^ For more detailed analysis, the angles between the polarization directions of the B atoms after full relaxation are plotted in Figure 4 for the ground-state, NoDW, 90DW and 180DW configurations, along with two configurations with the polarization directions of half of the B atoms rotated by 60° and 120° (60ROT and 120ROT, respectively) as initial structures, and the corresponding energies are summarized in Table S6. The energy of the 180DW configuration in PMN-33PT is similar to that in PbTiO_3_, while the energy of the 90DW configuration in PMN-33PT is much higher than that in PbTiO_3_.^56^ The polarization maps (Figure 4B–E) and energies among ground state, NoDW, 60ROT, and 120ROT are quite close to each other, indicating that the above-mentioned configurations can switch easily, in other words, there exist many dynamic atomistic polar domain walls with angles less than 35°, agreeing well with previous results in the literature.^8,50^

**Figure 4:**
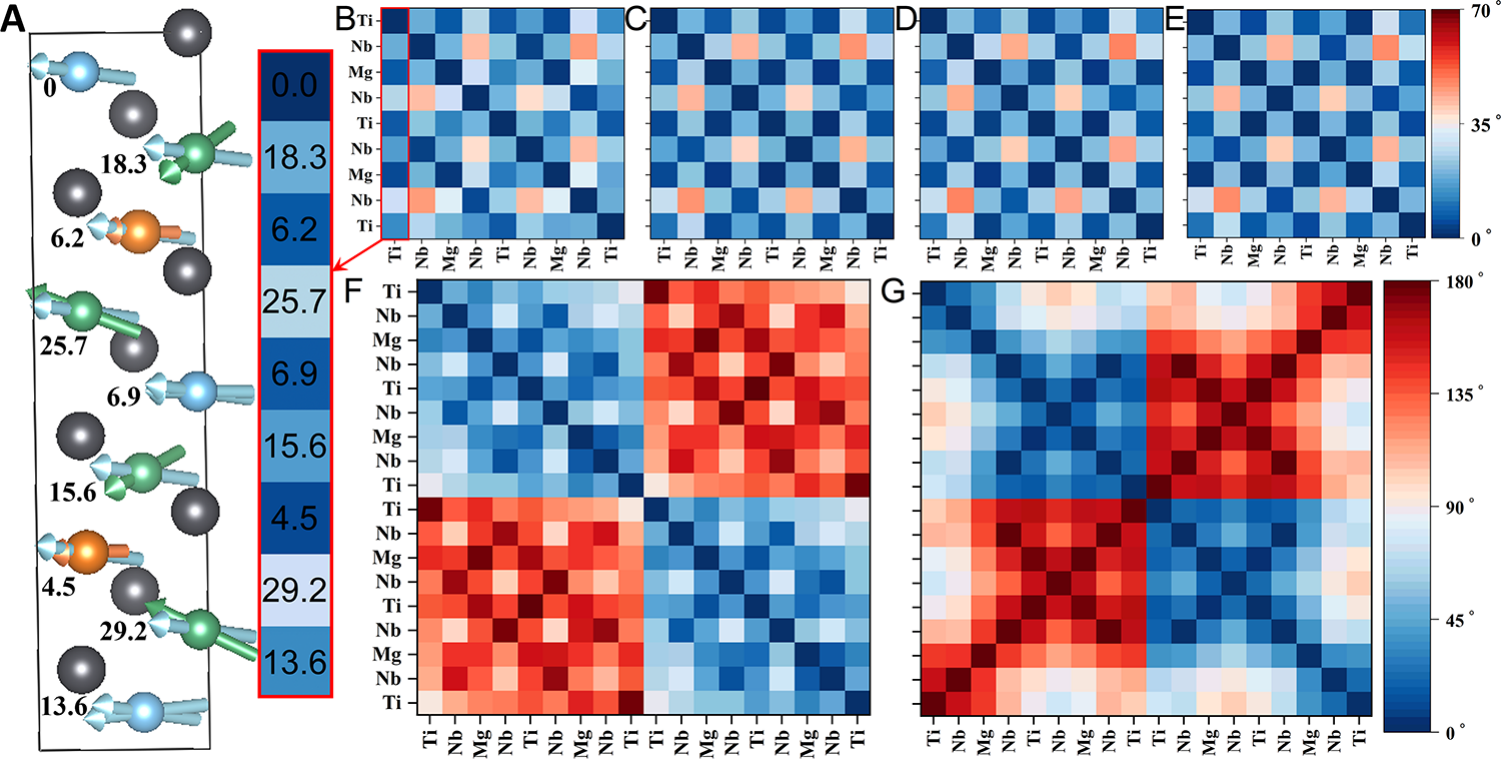
Angles between B-cation polarization directions obtained from Born effective charges for various configurations. (A) Polarization directions of B-site cations (blue = Ti, green = Nb and orange = Mg) with angles (degrees) with respect to polarization direction of the first Ti atom in the ground state; (B–G) polarization maps of (B) ground state; (C) NoDW supercell of the ground state; (D) 60ROT, (E) 120ROT, (F) 90DW, and (G) 180DW configurations.

In addition, the probabilities of dynamic atomistic polar structures and configurational entropy plotted in Figure S12, indicate a significant decrease of the probability of the no domain wall (NoDW) ground-state configuration above 180 K and down to 50% at 510 K, which is associated with increased probability of 60ROT and 120ROT configurations which are dynamic atomistic polar structures leading to extraordinary EO properties.

## 4 Conclusions

High-density transparent ferroelectric ceramics were prepared using SPS with subsequent sintering in an oxygen-rich atmosphere. The transparency is attributed to the wide band gap and the minimization of light scattering of defects such as pores, grain boundaries and domain walls. The ultrahigh linear EO coefficient with good thermal stability up to 57 °C, can be attributed to sensitive electronic polarization associated with dynamic atomistic polar structures located in switchable and thermally stable domains. This proposed theoretical model could serve as the basis of a tool for prediction of EO properties. Compared to piezoelectric single crystals, piezoelectric ceramics have advantages in terms of the processing cost of materials and accurate control of composition and properties. The transparent LPMN-33PT ceramic significantly outperforms single crystal counterparts. The new physical mechanism, on dynamic atomistic polar structures and high linear EO coefficient, provides guidelines for studies of low-cost transparent piezoelectric ceramic materials with very high linear EO coefficient values operating at low applied voltage.

## Supplementary Material



## Data Availability

Simulation data for the structure curation, relaxation, and polarization will be available at 10.5281/zenodo.8322450 upon publication. Other data that support the findings of this study are available on request from the corresponding authors.

## Notes

EO coefficient measurement; SEM images of LPMN-33PT bulk ceramic and powder; dielectric properties of LPMN-*x*PT ceramics; Polarization of LPMN-33PT ceramic; Crystal structure details for LPMN-33PT ceramic before and after poling; field induced strain of LPMN-xPT ceramics; TEM observations; ab initio electronic structure simulations; zentropy theory (PDF)
