# Supplementary material for: Dynamic Atomistic Polar Structure Underpins Ultrahigh Linear Electro-Optic Coefficient in Transparent Ferroelectric Ceramics
Source: J Am Chem Soc. 2025 Nov 4;147(46):42909–17. doi: 10.1021/jacs.5c15699 (PMC12636007; doi:10.1021/jacs.5c15699)
Supplement: Supplementary file 1 [file ja5c15699_si_001.pdf]

Supplementary information for

Dynamic atomistic polar structure underpins ultrahigh linear electro-optic coefficient in transparent ferroelectric ceramics

Qinghui Jiang[1]*, Weigang Zhao[2], Man Zhang[3,4], Jian-Ping Zhou[5], Mingqing Liao[6], Andriy Smolyanyuk[7], Zixuan Wu[4], Chenglong Jia[8], Xiaoyong Wei[2], Cedric Weber[9], Nadezda V. Tarakina[10], Isaac Abrahams[11]*, Jan M. Tomczak[12,7], Zi-Kui Liu[13], Vladimir Roddatis[14], and Haixue Yan[4]*

[1]State Key Laboratory of Materials Processing and Die and Mould Technology, and School of Materials Science and Engineering, Huazhong University of Science and Technology; Wuhan, 430074, PR China

[2]Electronic Materials Research Laboratory, Key Laboratory of the Ministry of Education & International Center for Dielectric Research, School of Electronic Science and Engineering, Xi'an Jiaotong University; Xi'an, 710049, PR China

[3]School of Mechanical Engineering, University of Leeds; Leeds, LS2 9JT, United Kingdom

[4]School of Engineering and Materials Science, Queen Mary University of London; London, E1 4NS, United Kingdom

[5]School of Physics and Information Technology, Shaanxi Normal University; Xi'an, 710119, PR China

[6]School of Materials Science and Engineering, Jiangsu University of Science and Technology; Zhenjiang, 212003, PR China

[7]Institute of Solid State Physics; TU Wien, 1040 Vienna, Austria

[8]School of Physical Science and Technology, Lanzhou University, Lanzhou 730000, PR China

[9]Quantum Brilliance Pty, The Australian National University; Canberra ACT 2600, Australia

[10]Max Planck Institute of Colloids and Interfaces Department of Colloid Chemistry; Potsdam, 14476, Germany

[11]Department of Chemistry, Queen Mary University of London; London, E1 4NS, United Kingdom

[12]Department of Physics, King's College London; London, WC2R 2LS, United Kingdom

[13]Department of Materials Science and Engineering, College of Earth and Mineral Science, The Pennsylvania State University; PA 16802, USA

[14] GFZ Helmholtz Centre for Geosciences; Telegrafenberg, D-14473 Potsdam, Germany

*Email: qhjiang@hust.edu.cn; i.abrahams@qmul.ac.uk; h.x.yan@qmul.ac.uk.

Table of Contents

1. EO coefficient measurement....4

Figure S1....4

Figure S2....6

Table S1....6

2. SEM images of LPMN-33PT bulk ceramic and powder....7

Figure S3....7

4. Dielectric properties of LPMN-*x*PT ceramics....9

Figure S4....9

5. Polarization of LPMN-33PT ceramic....10

Table S2....10

6. Crystal structure details for LPMN-33PT ceramic before and after poling....11

Figure S5....11

Table S3....12

Table S4....13

7. The field induced strain of LPMN-xPT ceramics....14

Figure S6....14

8. TEM observations....15

Figure S7....15

Figure S8....16

Figure S9....19

9. *ab initio* electronic structure simulations....20

Table S5....21

Figure S10........22
10. Zentropy theory........23
Figure S11........27
Table S6........28
Figure S12........29
Supplementary references........30

1. EO coefficient measurement

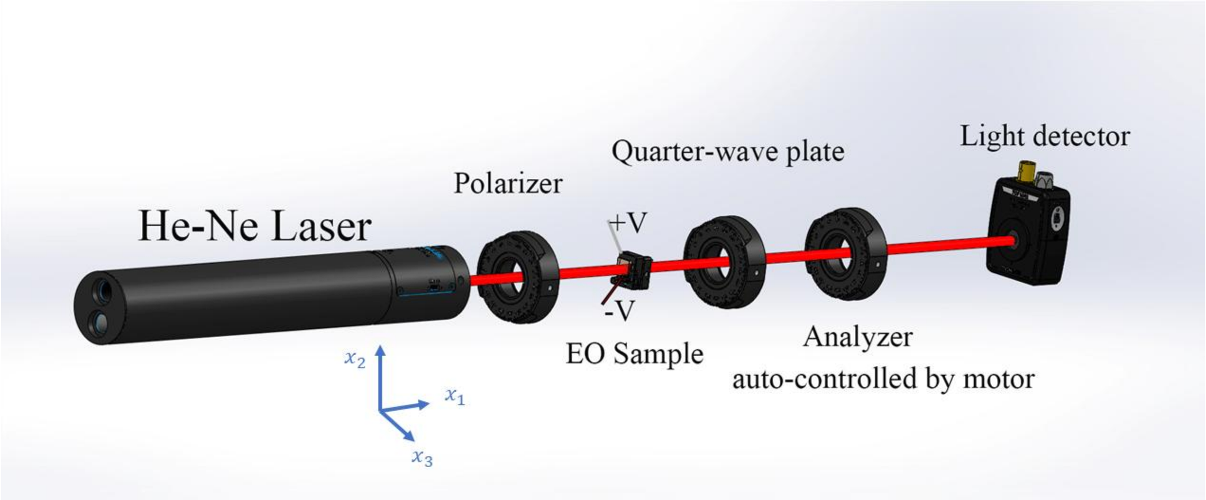

Figure S1. the minimum transmission point method to measure the effective EO coefficient.

The effective electro-optic coefficient (EOC, γ_c) of the samples was measured by the minimum-transmission point measurement method with a 633 nm He-Ne laser as the light source. As shown in Figure S1, a linear polarizer P was set with its polarization direction at 45° to the x_2 direction, the sample was placed between the linear polarizer P and a quarter-wave plate poled along the x_2 (vertical) direction. The fast axis of the quarter-wave plate was set at 45° to the x_2 direction, and the polarization direction of the analyzer was set as β to the x_2 direction. The actual phase shift φ_E is equal to twice the measured rotation angle $\beta_E = \Delta\beta$ (original data) by the analyzer.[1] In transverse mode, a dc electric field in the x_2- x_3 plane was applied along the poling direction, which was perpendicular to the beam. The EO properties of a sample cause a shift in the phase of polarized light with $\varphi_E = \frac{2\pi}{\lambda}\Delta nl = 2\beta_E$. The effective EOC can then be expressed as Equation S1:

$$\gamma_c = \frac{\lambda d}{\pi l n^3} \times \frac{\varphi_E}{U} = \frac{\lambda}{\pi l n^3} \times \frac{\varphi_E}{\frac{U}{d}} = \frac{\lambda}{\pi l n^3} \times \frac{2\beta_E}{E} \qquad \text{(S1)}$$

where, *d* is the distance between the two electrodes on the sample, *l* is the thickness of the sample along the light path and λ is the wavelength of the laser (633 × 10^{-9} m). In the main text, the thickness of LPMN-*x*PT samples is 0.16 mm and phase shift $\varphi_E = 2(\beta_E)$ is used in the figures and descriptions. Refractive indices of PMN-*x*PT in Table S1 are used as values of *n*.

Figure S2 shows original data (rotation angle (β_E) – applied field(E)) from a commercial $LiNbO_3$ crystal (Hengguang Photoelectric Ltd., China) as a reference material.[2] The linear EO coefficient of $LiNbO_3$ can be calculated as 21 pm/V with details below, which is consistent with the value previously reported.[3]

$$\gamma_c = \frac{\lambda}{\pi l n^3} \times \frac{2\beta_E}{E} = \frac{2 \times 633 \times 10^{-9}}{\pi \times 0.16 \times 10^{-3} \times 2.203^3} \times 0.00882 \times 10^{-5} \approx 21\, pm/V$$

where l is the thickness of the sample (0.16 mm) along the light path, λ is the wavelength of laser (633 × 10^{-9} m), *n* of $LiNbO_3$ is 2.203, and $\frac{\beta_E}{E}$ = 0.00882 $\times 10^{-5}$ m/V from Fig S2.

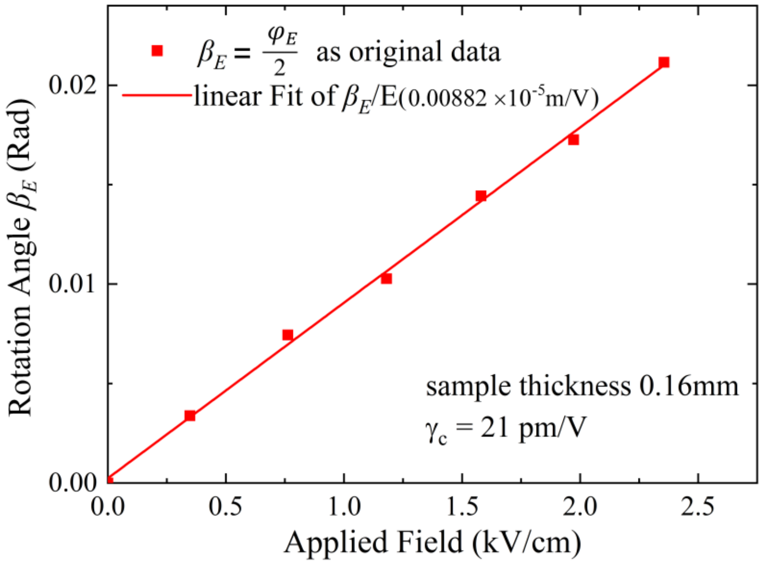

Figure S2. Linear EO effect in $LiNbO_3$ commercial single crystal as a reference material.

Table S1. Reflection loss and theoretical transparency values of LPMN-*x*PT ceramics calculated from refractive indices.

Composition	Refractive index	Reflection loss	Theor. transparency (%)
LPMN-25PT	2.58[4]	0.3261	67.40
LPMN-29PT	2.59[4]	0.3280	67.20
LPMN-33PT	2.60[4]	0.3299	67.01
LPMN-36PT	2.61[4]	0.3318	66.82

Note: The reflection loss at two faces of the pellet can be calculated according to the Fresnel equations.

2. SEM images of LPMN-33PT bulk ceramic and powder.

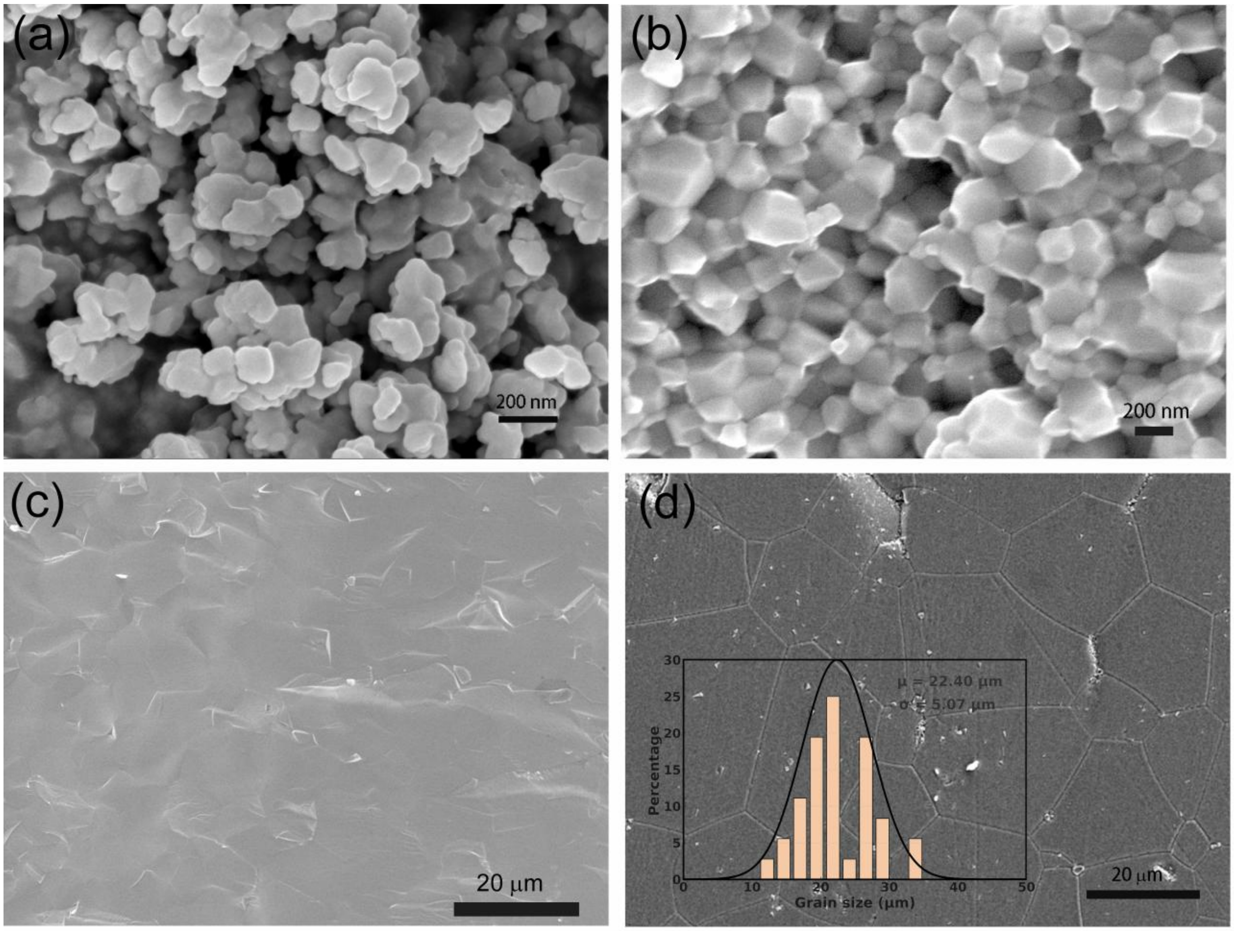

Figure S3. SEM images of LPMN-33PT ceramic powder and bulk samples. (a) Powder sample after high-energy ball milling, (b) fracture surface of a bulk ceramic sample consolidated by SPS at 950 °C, (c) fracture surface of a bulk ceramic sample after annealing at 1200 °C, and (d) thermally etched (900 °C and 15 mins) surface of a bulk ceramic sample annealed at 1200 °C.

Figure S3 shows SEM images of LPMN-33PT ceramic powder (a) and bulk samples (b-d). The fracture surface of the CAPAD prepared bulk ceramic has high density and the grain size is less than a half micron. Figure S3c is the fracture surface of a bulk ceramic with high density after a second annealing step. The fracture damage includes

transcrystalline rupture, making it difficult to distinguish the grain size information. Grain boundaries are more clearly seen in the SEM image (Figure S3d) of a polished bulk ceramic sample which was thermally etched at 900 °C for 15 minutes, revealing an average grain size of ~22 μm through grain size statistics.

3. Dielectric properties of LPMN-*x*PT ceramics.

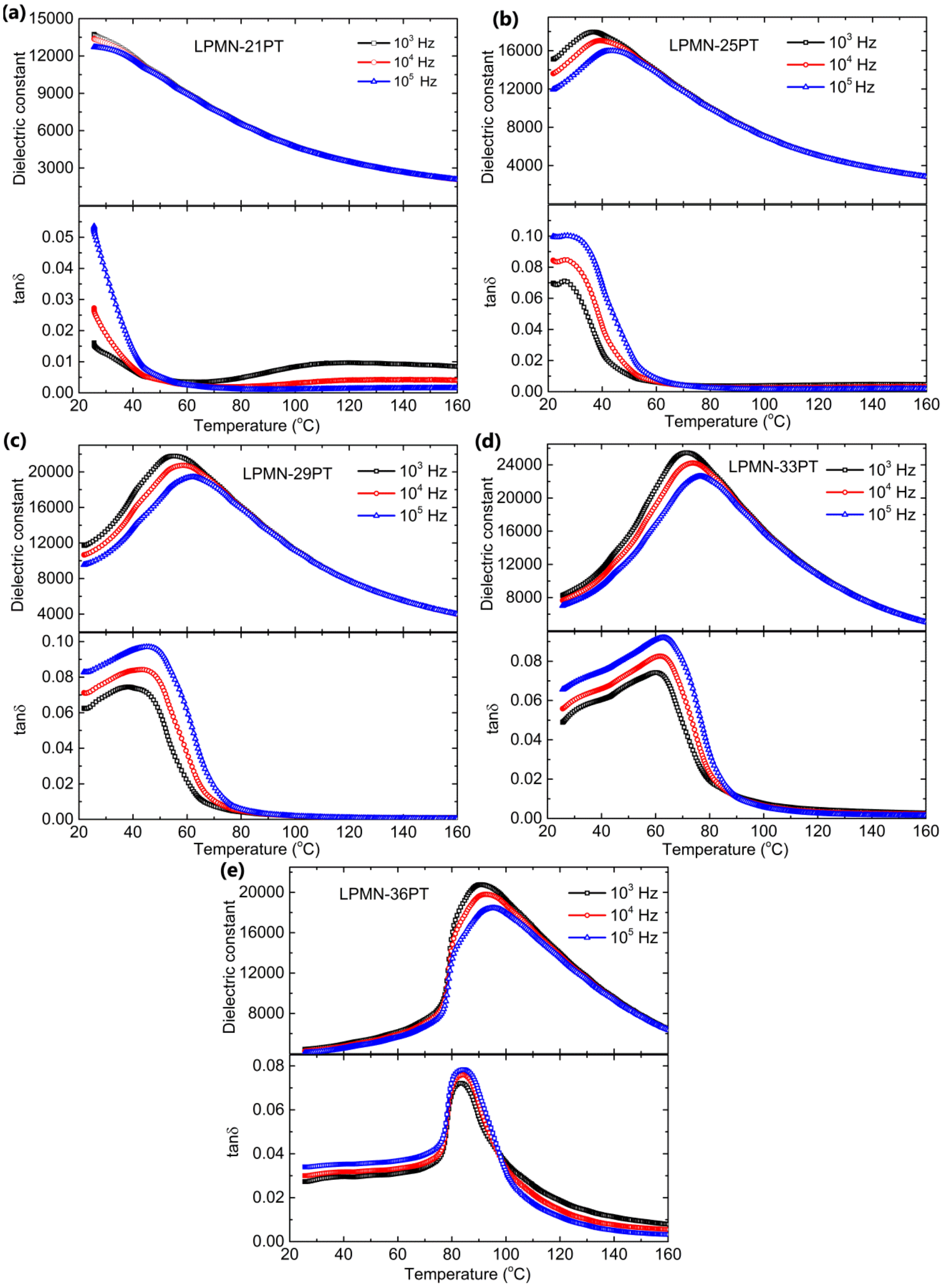

Figure S4. Temperature-dependent dielectric permittivity and loss curves of LPMN-*x*PT unpoled ceramics for *x* = (a) 21, (b) 25, (c) 29, (d) 33 and (e) 36 compositions.

4. Polarization of LPMN-33PT ceramic

Table S2. Piezoelectric coefficient, d_{33}, values for LPMN-33PT after DC poling.

Poling condition	0.4 kV/cm (15 min, DC)	0.8 kV/cm (15 min, DC)	1.2 kV/cm (15 min, DC)	1.8 kV/cm (15 min, DC)	4 kV/cm (30 min, DC)
d_{33}	20 pC/N	49 pC/N	82 pC/N	305 pC/N	1450 pC/N

5. Crystal structure details for LPMN-33PT ceramic before and after poling

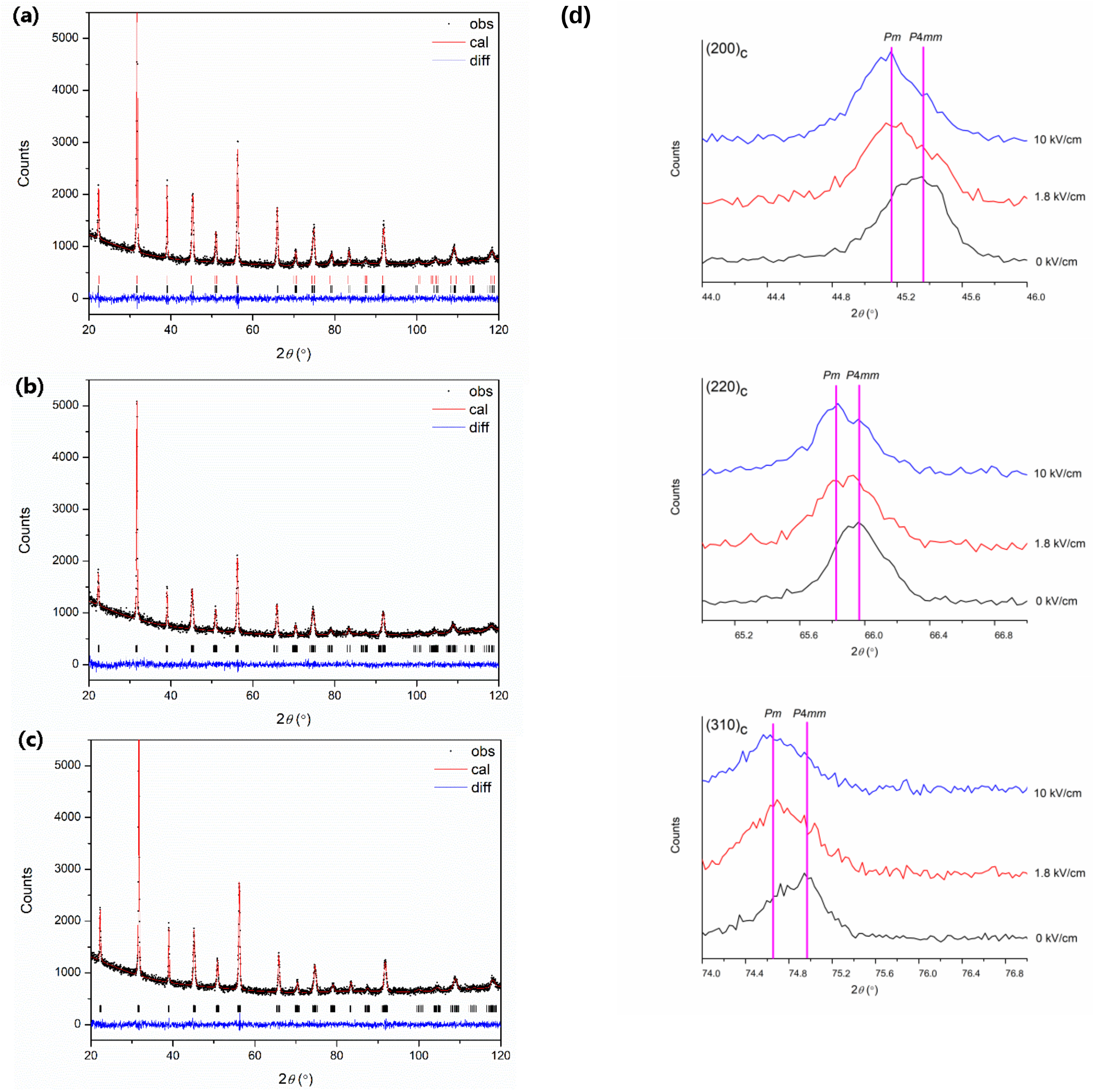

Figure S5. Fitted X-ray powder diffraction patterns for LPMN-33PT ceramic. (a) Unpoled, (b) poled at 1.8 kV/cm and (c) poled at 10 kV/cm. Reflection positions are indicated by markers for *Pm* (black) and *P4mm* (red) phases. (d) Changes in selected pseudo-cubic reflections of LPMN-33PT ceramic on poling.

Table S3. Crystal and Refinement Parameters for LPMN-33PT prior to and after poling

	**Unpoled**	**Poled at 1.8 kV/cm**	**Poled at 10 kV/cm**
**Phase 1**			
Name	Lead lanthanum magnesium titanium niobate		
Empirical formula	$Pb_{0.9625}La_{0.025}Ti_{0.33}Nb_{0.44}Mg_{0.22}O_3$		
Formula weight	313.56 g/mol		
Crystal system	Monoclinic		
Space group	*Pm*		
Unit cell dimensions	*a* = 4.0303(5) Å<br>*b* = 4.0160(5) Å<br>*c* = 4.0205(5) Å<br>β = 89.83(1)°	*a* = 4.0451(8) Å<br>*b* = 4.0126(9) Å<br>*c* = 4.0304(7) Å<br>β = 89.60(2)°	*a* = 4.0450(7) Å<br>*b* = 4.0079(6) Å<br>*c* = 4.0300(5) Å<br>β = 89.66(1)°
Volume	65.07(1) $Å^3$	65.42(3) $Å^3$	65.33(1) $Å^3$
Z	1	1	1
Density (calc)	8.001 g/cm^3	7.959 g/cm^3	7.973 g/cm^3
Weight fraction	0.33(3)	1.0	1.0
**Phase 2**			
Name	Lead lanthanum magnesium titanium niobate		
Empirical formula	$Pb_{0.9625}La_{0.025}Ti_{0.33}$<br>$Nb_{0.44}Mg_{0.22}O_3$		
Formula weight	313.56 g/mol		
Crystal system	Tetragonal		
Space group	*P*4*mm*		
Unit cell dimensions	*a* = 4.0154(5) Å<br>*c* = 4.0456(8) Å		
Volume	65.23(2) $Å^3$		
Z	1		
Density (calc)	7.982 g/cm^3		
Weight fraction	0.67(1)		
No. of observations/ restraints/parameters	3172/0/55	3172/0/49	3172/0/49
No. of reflections used	324	236	234
Final R-factors	R_p = 0.0344,<br>R_{wp} = 0.0434<br>R_{ex} = 0.0352,<br>χ^2 = 1.549	R_p = 0.0354,<br>R_{wp} = 0.0448<br>R_{ex} = 0.0371,<br>χ^2 = 1.481	R_p = 0.0339,<br>R_{wp} = 0.0426<br>R_{ex} = 0.0354,<br>χ^2 = 1.474
Maximum atomic shift	0.00 Å	0.00 Å	0.01 Å

Table S4.

Atomic Coordinates and Isotropic Thermal Parameters for LPMN-33PT prior to and after poling

(a) Unpoled Phase 1 (monoclinic)

**Atom**	**Site**	***x***	***y***	***z***	**Occ.**	U_{iso} **(Å^2)**
Pb	1a	0	0	0	0.9625	0.024(1)
La	1a	0	0	0	0.025	0.024(1)
Ti	1b	0.467(5)	0.5	0.532(4)	0.333	0.02
Nb	1b	0.467(5)	0.5	0.532(4)	0.444	0.02
Mg	1b	0.467(5)	0.5	0.532(4)	0.222	0.02
O1	1a	0.4970	0	0.5240	1	0.02
O2	1b	0.5070	0.5	0.0090	1	0.02
O3	1b	-0.0460	0.5	0.5790	1	0.02

(b) Unpoled Phase 2 (tetragonal)

**Atom**	**Site**	***x***	***y***	***z***	**Occ.**	U_{iso} **(Å^2)**
Pb	1a	0	0	0	0.9625	0.024(1)
La	1a	0	0	0	0.025	0.024(1)
Ti	1b	0.5	0.5	0.529(6)	0.333	0.02
Nb	1b	0.5	0.5	0.529(6)	0.444	0.02
Mg	1b	0.5	0.5	0.529(6)	0.222	0.02
O1	1b	0.5	0.5	0.054	1	0.02
O2	2c	0.5	0.0	0.601	1	0.02

(c) Poled at 1.8 kV/cm (monoclinic)

**Atom**	**Site**	***x***	***y***	***z***	**Occ.**	U_{iso} **(Å^2)**
Pb	1a	0	0	0	0.9625	0.027(2)
La	1a	0	0	0	0.025	0.027(2)
Ti	1b	0.498(4)	0.5	0.517(6)	0.333	0.02
Nb	1b	0.498(4)	0.5	0.517(6)	0.444	0.02
Mg	1b	0.498(4)	0.5	0.517(6)	0.222	0.02
O1	1a	0.4970	0	0.5240	1	0.02
O2	1b	0.5070	0.5	0.0090	1	0.02
O3	1b	-0.0460	0.5	0.5790	1	0.02

(d) Poled at 10 kV/cm (monoclinic)

**Atom**	**Site**	***x***	***y***	***z***	**Occ.**	U_{iso} **(Å^2)**
Pb	1a	0	0	0	0.9625	0.026(1)
La	1a	0	0	0	0.025	0.026(1)
Ti	1b	0.473(7)	0.5	0.487(12)	0.333	0.02
Nb	1b	0.473(7)	0.5	0.487(12)	0.444	0.02
Mg	1b	0.473(7)	0.5	0.487(12)	0.222	0.02
O1	1a	0.4970	0	0.5240	1	0.02
O2	1b	0.5070	0.5	0.0090	1	0.02
O3	1b	-0.0460	0.5	0.5790	1	0.02

6. The field induced strain of LPMN-*x*PT ceramics

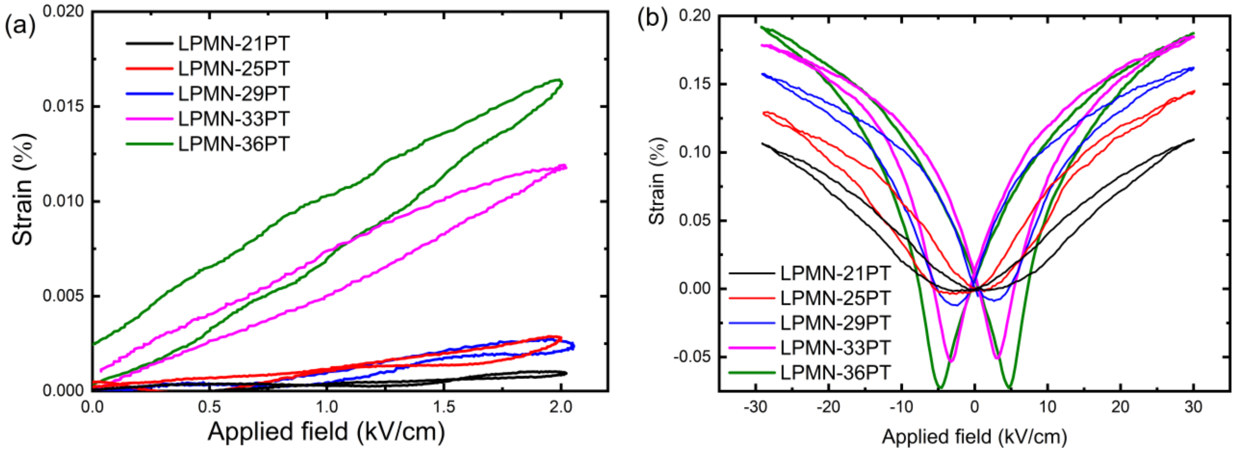

Figure S6. Unipolar (a) and bipolar (b) strain at room temperature for LPMN-xPT samples

7. TEM observations

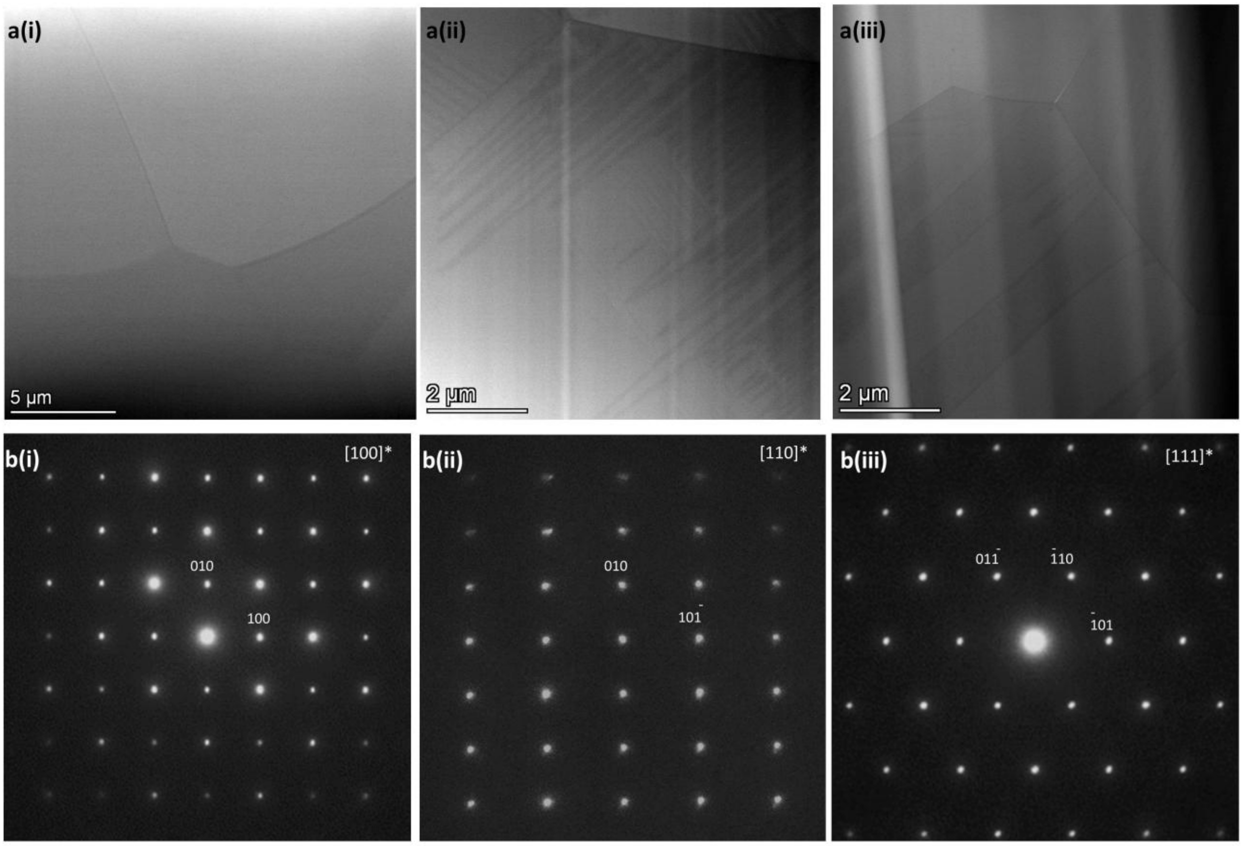

Figure S7. Domains and local polar regions in transparent LPMN-*x*PT ceramics. (a) low magnification HAADF images of LPMN-29PT **(i)**, LPMN-33PT **(ii)**, and LPMN-36PT **(iii)** (the vertical lines are due to the curtain effect of FIB preparation); (**b)** SAED patterns collected along [100] **(i)**, [110] **(ii)** and [111] **(iii)** zone axes for LPMN-33PT, no additional spots due to the presence of other phases or defects are visible.

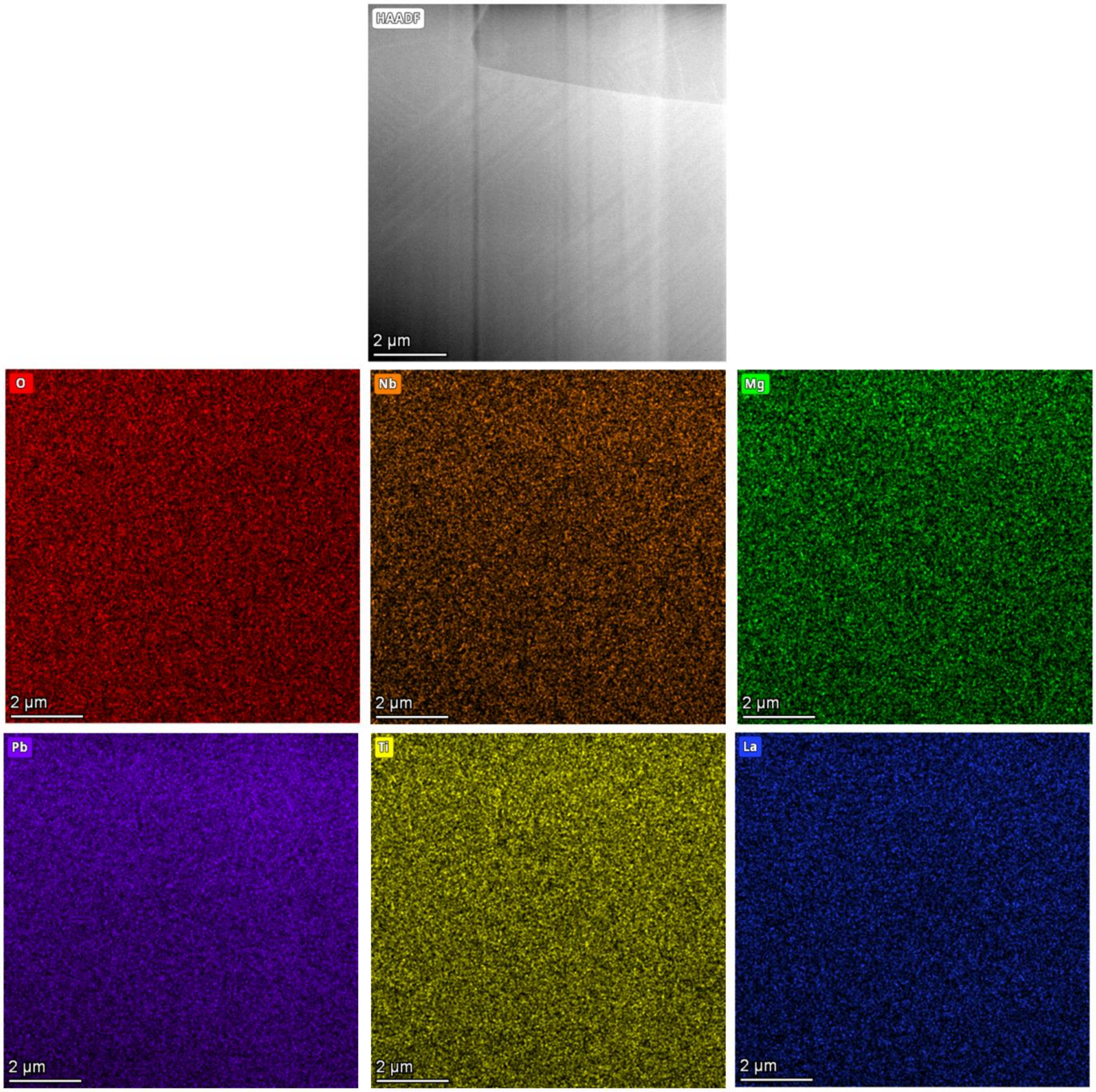

Figure S8. Elemental distribution chemical maps collected from the LPMN-33PT specimen. No contrast variation at the interfaces between adjacent grains or lamella-shaped domains is observed.

Low magnification Scanning Moiré Fringes (SMF)[5] in a limited part (~100 nm in the direction of grain bending) of a whole image evidences a remarkable curvature of the grain (Figure S9). Interestingly, HR-STEM imaging with atomic resolution of areas much larger than the area of SMF did not reveal the presence of defects (dislocations, etc.). This means that changes of atomic structure are very small for each individual unit cell, and the observed domains occur due to numerous collective unit cell distortions. This is somewhat in contrast to reported observations of polar nanodomains, which are of a few nanometers in size only[6-8]. However, in this case these domains have to have a cylindrical or ellipsoidal morphology, otherwise they would overlap and potentially be invisible. Modern state-of-the-art microscopes equipped with a corrector of spherical aberration at the probe side have a small depth of focus.[9] Thus, high resolution STEM images could be insensitive to the structural features of elongated objects along the beam propagation. If polar nanodomains do exist, it can be assumed that PMN-*x*PT ferroelectrics accommodate the morphology of domains to the geometrical constraints/size of the studied object. Thus, in thin areas the polar nanodomains are observed, while with increasing ceramic thickness, lamella-like and bulk[10] domains are visible.

For thickness evaluation, an analysis of the zero-loss peak of electron energy loss spectroscopy was used. Taking an experimentally measured averaged chemical composition and using a mean free path estimator script,[11-12] the estimated thickness of the area imaged in Figure S9 is about 80 nm.

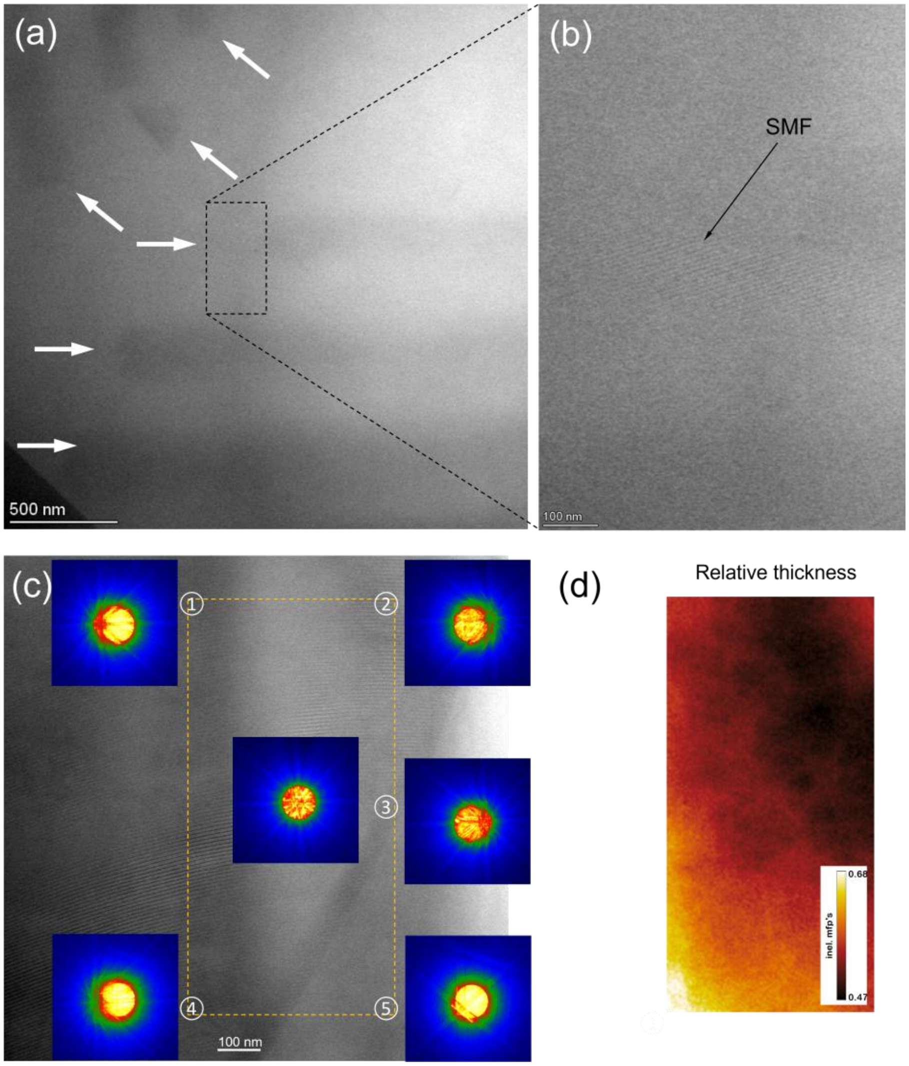

Figure S9. STEM images of LPMN-33PT ceramic. (a) A low magnification image showing ferroelectric domains marked by white arrows; (b) An enlarged central part of the STEM image shown in (a) with Scanning Moiré Fringes (SMF); (c) An example of significantly curved area of 500 nm x 1000 nm the LPNM-33PT grain. The difference of orientation is about 2 degrees; (d) Corresponding relative thickness map.

8. *ab initio* electronic structure simulations

To assess the stability of the field-induced structural changes in LPMN-33PT prior to and after poling, *ab initio* electronic structure simulations were performed. Technical details and methods employed are described in Section 2.8 of the article. Here, in Table S5, we collect the results for the obtained total energies and electrical polarizations. The initial (unrelaxed) disorder configurations were first ranked by their total E_Γ obtained with Γ-point sampling (Brillouin zone approximated with single k-point) with VASP. Leading structures were then internally optimized with WIEN2k yielding total energies E_{tot}. Note that, in WIEN2k, the origin of energy is not absolute but influenced in particular by the atomic sphere radii, which is why E_Γ and E_{tot} are not directly comparable. Next, the spontaneous electrical polarization vector (P_1, P_2, P_3) of the relaxed poled structures was computed with respect to the unpoled reference using BerryPI. Note that owing to the non-orthogonal basis vectors of the unit-cells, the polarizations P_i (I = 1, 2, 3) do not directly correspond to P_α (α = x, y, z) in cartesian directions. The finite polarization indicates that in the atomic optimization, the poled structure does not relax to the unpoled structure, indicating the former to be at least meta-stable. The poling-induced polarization is illustrated in Figure S10 for the disorder configuration #5319.

Table S5. Electric polarization and total energies of disordered configurations mimicking PMN-33PT

Pm conf.	Structure	P_1 [C/m^2]	P_2 [C/m^2]	P_3 [C/m^2]	E_Γ [eV]	E_{tot} [Ry]
#5319	Poled	-0.042	-0.441	-0.104	-324.26	-416998.191
	Unpoled	0.083	-0.114	0.196	-315.63	-416998.188
	Difference	-0.124	-0.330	-0.300	-8.63	-0.003
#5511	Poled	-0.037	-0.362	-0.047	-324.31	-416998.191
	Unpoled	0.087	-0.140	0.234	-315.70	-416998.193
	Difference	-0.123	-0.221	-0.281	-8.61	0.002
#5546	Poled	-0.044	-0.024	0.072	-323.92	-416998.189
	Unpoled	-0.070	-0.027	0.142	-315.31	-416998.195
	Difference	0.025	0.003	-0.070	-8.61	0.006

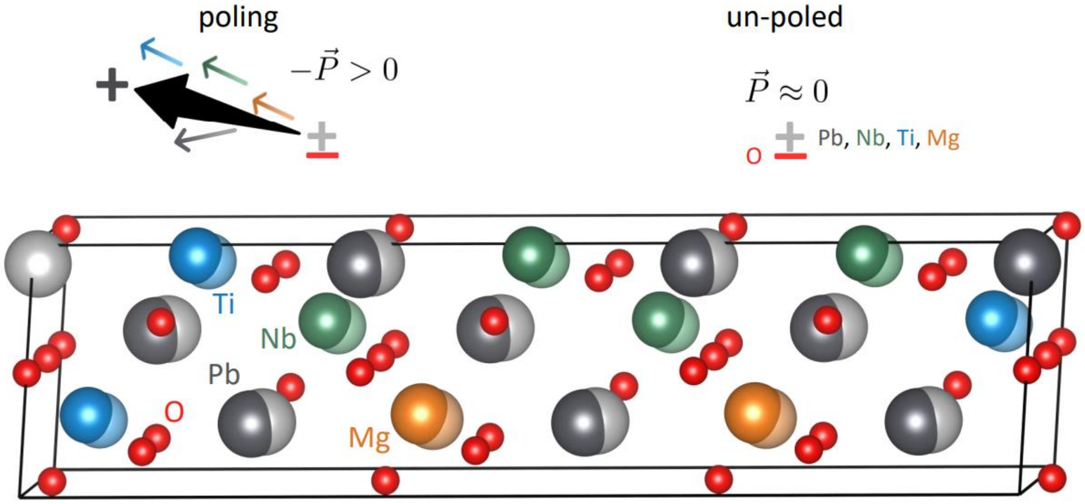

Figure S10. Structural impact of the poling field. Light (dark) shaded atoms indicate positions before (after) poling for the disorder configuration #5319. While oxygen anions ("-") remain virtually fixed, Pb, Nb, Ti, Mg cations ("+") are displaced in directions (schematically indicated by colored arrows) that yield a net polarization vector $\vec{P}$ (schematically indicated by the large black arrow).

10. Zentropy theory

10.1 Introduction to zentropy theory

The thermodynamics of ferroelectric materials and their phase transitions, i.e., ferroelectric to paraelectric (FE-PE) transitions, are commonly described by the phenomenological Landau-Ginsburg-Devonshire theory (LGDT) and more recently by effective Hamiltonian and various potentials, all with model parameters fitted to experimental or theoretical data. Even though the LGDT formalism describes phase transition behavior well macroscopically, it does not correctly describe the microscopic features, such as the domain walls (DWs) and local non-vanishing polarizations in the macroscopic cubic structure at high temperatures.[13-17] The predictions from effective Hamiltonian approaches presented a remarkable agreement with experimental observations in terms of the phase stability sequence, providing insights into the order-disorder versus displacive character of the transitions and the importance of various interaction terms in the effective Hamiltonians.[17-19] However, the fitting of the energy surface, in terms of a Taylor expansion, reduces the quantitative predictability of transition temperatures. In accordance with the statistical mechanics developed by Gibbs,[20] Liu's group developed the zentropy theory that considers a macroscopically homogeneous phase at finite temperatures with a set of configurations that the system experiences statistically with the same external constraints such as temperature, volume/strain, and electric field as the system.[21-24]

Two major challenges are how to select the configurations that the system experiences and calculate their free energies as a function of external constraints. In the zentropy theory,

one starts from the ground-state configuration of a system at zero K which has the lowest energy and is defined by a unique electron density distribution based on the density functional theory (DFT).[25-26] By exhausting all internal degrees of freedom, **ergodic** symmetry-breaking non-ground-state configurations are explored with their free energies predicted from DFT-based first-principles calculations. Liu et al.[23] applied the zentropy theory to $PbTiO_3$, where the ground-state configuration is tetragonal with polarization, and the non-ground-state configurations are tetragonal with polarization and 90 or 180 degree domain walls, in accordance with the experimental local structure analysis[13, 27-28] and *ab initio* molecular dynamic simulations in the literature.[29] With the domain wall energies predicted by DFT in the literature and the assumption of equal entropy for all three configurations, the parameter-free prediction of the FE-PE transition of $PbTiO_3$ under ambient pressure shows remarkable agreement with experimental observations.[30]

10.2 Application of zentropy theory to the PMN-33PT system

The PMN-33PT system is more complicated than $PbTiO_3$ due to the complex multicomponent interactions. As discussed in Section 2.8 in the article, the ground-state configuration of the present PMN-33PT system was found to be No. 5511 as shown in Table S5 with the general polarization along [011]. The polarization supercell with B-cations shifted slightly with a scale matrix of [[1, 0, -1], [0, 1, 0], [1, 0, 1]] (to make the supercell close to orthogonal) based on the 5511 structure is shown in Figure S11a and is referred to as the NoDW ground-state configuration. The fully relaxed structure has an orthorhombic-like

crystal structure. The polarization was obtained by means of the Born effective charge, as Equation S2,[31]

$$\mathbf{P}_i = \frac{e}{\Omega}\mathbf{Z}_i \delta \mathbf{u}_i \tag{S2}$$

where $\mathbf{P}_i$, $\mathbf{Z}_i$, $\delta\mathbf{u}_i$ and Ω are the polarization, Born effective charge tensor, relative (to centrosymmetric structure) displacement of ion *i*, and the volume of the unit cell. The total polarization is the sum over all atoms.

10.3 Results and discussion

The polarization details of each unit cell (ABO_3, 5 atoms) are shown in Figure S11a1-a9 in the red-dashed rectangle. The total polarization is about 57.3 μC/cm^2, which is comparable with previous results of 62.4 μC/cm^2 and 43.5 μC/cm^2 for PMN-25PT.[32-33] Clearly, the polarization (displacement) of the O atoms is random in accordance with our experiments (Figure 2c). After relaxing, Ti5 and all Mg cations move along [011], while the other B-site cations move along [011] with tilts to [001] (Nb2, Nb6 and Ti9) and [010] (Nb4, Nb8 and Ti1), which agrees well with the results for PMN-35PT.[34] For A-site cations, Pb atoms move along the [011] direction with varying degrees of tilt. As pointed out by Otoničar et al.,[34] the Pb off-center displacement plays a key role in the relaxor behavior in relaxor-ferroelectric materials. We analyzed the lattice strain measured by Pb off-center displacement with the method used by Otoničar et al.[34] and extended this to three dimensions, as shown in Figure S11b. The lattice strain in the unit cell around the Ti cluster (Ti1 and Ti9) is quite small. The lattice strain (0.83) in the current system, located between PMN-30PT (0.96) and PMN-35PT (0.67), confirms the considerable lattice strain in the

current system. To link the local polar structure to properties, Liu et al.[35] proposed that the key is to balance the local polar disorder and polar length to achieve high piezoelectricity. PMN-33PT shows reasonable polar length and Pb disorder, and hence shows high piezoelectricity.

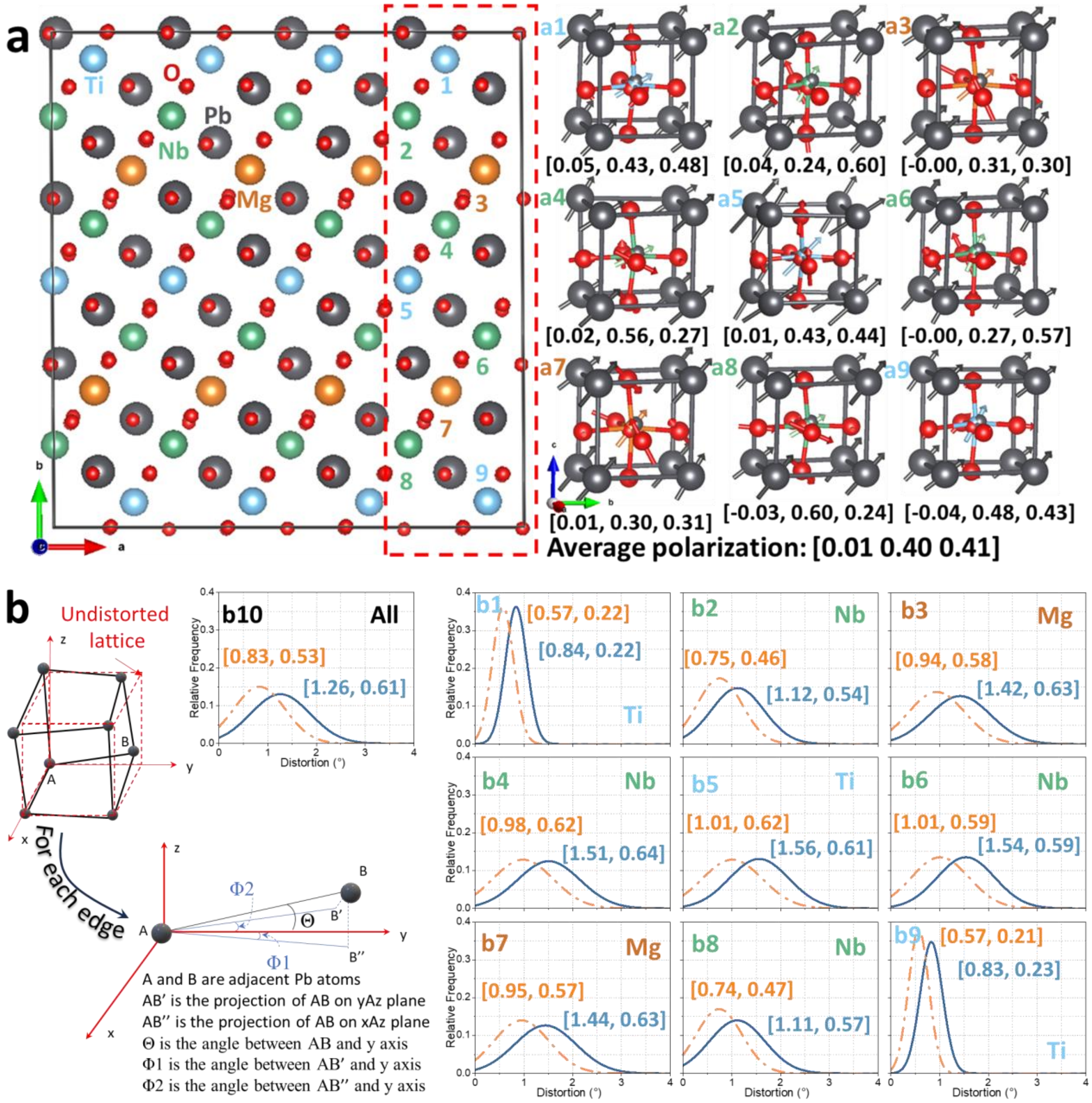

Figure S11. Atomic contribution to polarization in PMN-33PT. (**a**) Supercell (180 atoms in the cell with a supercell matrix of [[1, 0, -1], [0, 1, 0], [1, 0, 1]] based on structure 5511) of the optimized structure of PMN-33PT and the polarization contribution of each unit cell (5 atoms). Sub-figures a1-a9 show the polarization direction (vector direction) and relative value (vector length) of each unit cell in the red-dashed rectangle. To show the polarization clearly, the vectors of all O (Mg) atoms are zoomed in with a factor of 2 (3). The total polarization of each unit cell is calculated as $P_{uc} = P_B + 1/8 \times \mathrm{sum}(P_{Pb}) + ½ \times$

sum(P_O) and listed under each unit cell. The small red (gray) ball in the center of the unit cell is the centroid of the surrounding 6 (8) oxygen (lead) atoms. (**b**) Distortion of the unit cell. The distortion is defined as the angle between the Pb-Pb bond and the closest coordinate axis (y-axis in the schematic diagram). The probability density of the data is fitted to a normal distribution, and the fitted result [μ, σ] and relative frequency plot are shown in the sub-figures b1-b10. The solid and dash-dot lines are the angle in three dimensions (Θ) and the projection to the coordinate axis plane (Φ1 and Φ2), respectively.

The multiplicity and energy differences for NoDW, 90DW, 180DW, 60ROT, and 120ROT configurations are summarized in Table S6. Based on these data, the probabilities of these configurations and their entropy are plotted in Figure S12 using zentropy theory, representing the variation of dynamic atomistic polar structures as a function of temperature.[30]

Table S6. Multiplicity, domain wall (DW) energy, and total energy difference of different configurations with reference to NoDW in Figure 4.

	NoDW	90DW	180DW	60ROT	120ROT
**Multiplicity**	24	48	24	96	96
**DW energy, mJ/m^2**	N/A	121	133	N/A	N/A
**Energy, eV/Supercell**	0.000	0.209	0.877	0.090	0.094
**Energy,$\times 10^{-20}$J/Supercell**	0.000	3.349	14.050	1.435	1.511

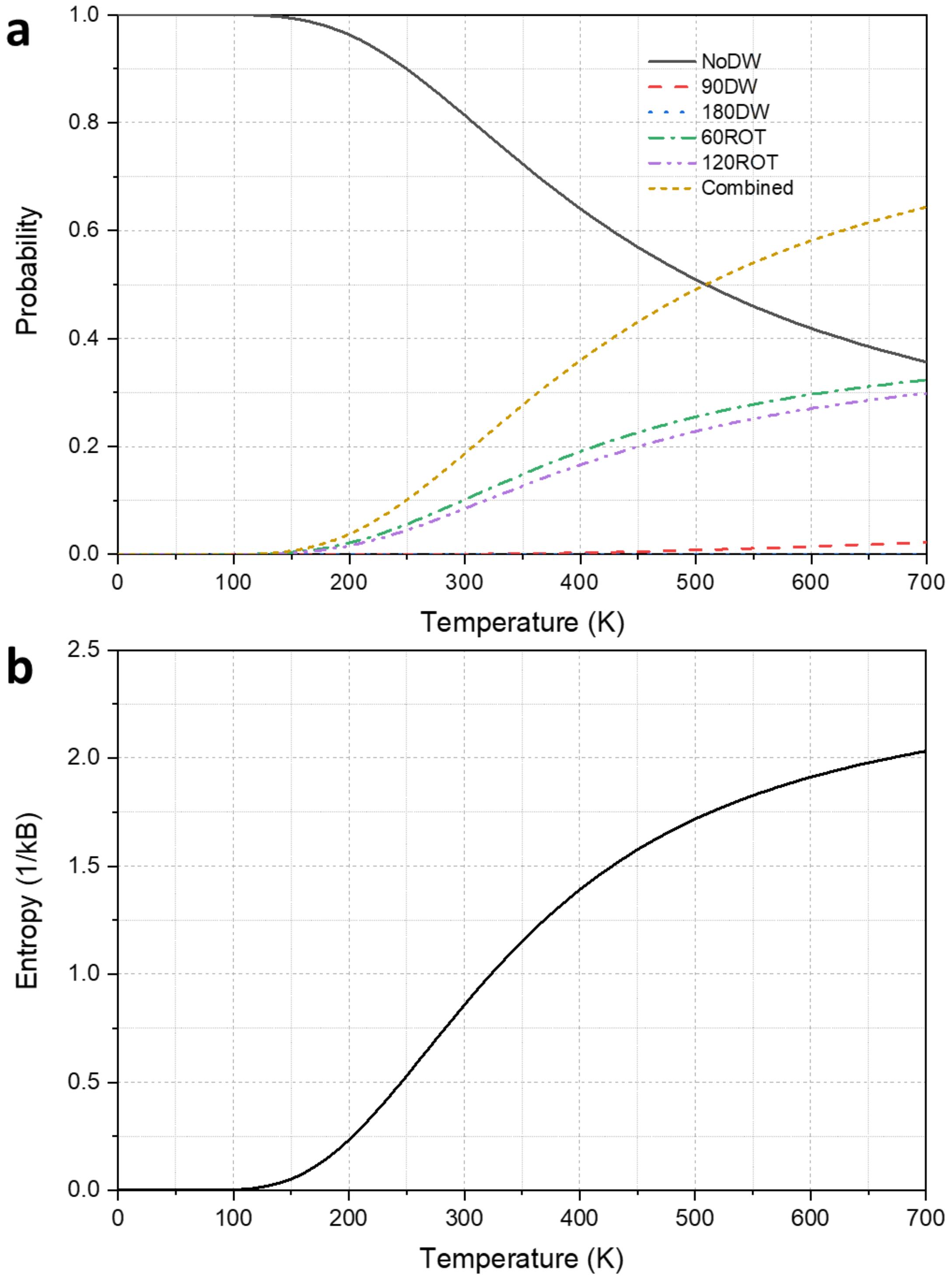

Figure S12. (a) Probabilities of various configurations as a function of temperature, and (b) the entropy of combined configurations.

Supplementary References

1. Aillerie, M.; Théofanous, N.; Fontana, M. D., Measurement of the electro-optic coefficients: description and comparison of the experimental techniques. *Applied Physics B* **2000,** *70* (3), 317-334.
2. Zhao, W. G.; Chen, H.; Fu, X. T.; Qiu, F. S.; Wei, X. Y.; Xu, Z., Single-path electro-optic coefficients measurement approach using multiple reflection interference. *Applied Optics* **2021,** *60* (33), 10372-10376.
3. Aillerie, M.; Fontana, M. D.; Abdi, F.; Carabatos-Nedelec, C.; Theofanous, N.; Alexakis, G., Influence of the temperature-dependent spontaneous birefringence in the electro-optic measurements of $LiNbO_3$. *Journal of Applied Physics* **1989,** *65* (6), 2406-2408.
4. Wan, X.; Luo, H.; Zhao, X.; Wang, D. Y.; Chan, H. L. W.; Choy, C. L., Refractive indices and linear electro-optic properties of $(1-x)Pb(Mg_{1/3}Nb_{2/3})O_3-xPbTiO_3$ single crystals. *Applied Physics Letters* **2004,** *85* (22), 5233-5235.
5. Su, D.; Zhu, Y. M., Scanning moire fringe imaging by scanning transmission electron microscopy. *Ultramicroscopy* **2010,** *110* (3), 229-233.
6. Kumar, A.; Baker, J. N.; Bowes, P. C.; Cabral, M. J.; Zhang, S.; Dickey, E. C.; Irving, D. L.; LeBeau, J. M., Atomic-resolution electron microscopy of nanoscale local structure in lead-based relaxor ferroelectrics. *Nature Materials* **2021,** *20* (1), 62-67.
7. Bencan, A.; Oveisi, E.; Hashemizadeh, S.; Veerapandiyan, V. K.; Hoshina, T.; Rojac, T.; Deluca, M.; Drazic, G.; Damjanovic, D., Atomic scale symmetry and polar nanoclusters in the paraelectric phase of ferroelectric materials. *Nature Communications* **2021,** *12* (1), 3509.
8. Geng, H.; Zeng, K.; Wang, B.; Wang, J.; Fu, Z.; Xu, F.; Zhang, S.; Luo, H.; Viehland, D.; Guo, Y., Giant electric field–induced strain in lead-free piezoceramics. *Science* **2022,** *378* (6624), 1125-1130.
9. Borisevich, A. Y.; Lupini, A. R.; Pennycook, S. J., Depth sectioning with the aberration-corrected scanning transmission electron microscope. *Proceedings of the National Academy of Sciences of the United States of America* **2006,** *103* (9), 3044-3048.
10. Liu, X.; Tan, P.; Ma, X.; Wang, D.; Jin, X.; Liu, Y.; Xu, B.; Qiao, L.; Qiu, C.; Wang, B.; Zhao, W.; Wei, C.; Song, K.; Guo, H.; Li, X.; Li, S.; Wei, X.; Chen, L.-Q.; Xu, Z.; Li, F.; Tian, H.; Zhang, S., Ferroelectric crystals with giant electro-optic property enabling ultracompact Q-switches. *Science* **2022,** *376* (6591), 371-377.
11. Malis, T.; Cheng, S. C.; Egerton, R. F., EELS log-ratio technique for specimen-thickness measurement in the TEM. *Journal of electron microscopy technique* **1988,** *8* (2), 193-200.
12. Iakoubovskii, K.; Mitsuishi, K.; Nakayama, Y.; Furuya, K., Thickness measurements with electron energy loss spectroscopy. *Microscopy Research and Technique* **2008,** *71* (8), 626-631.
13. Sicron, N.; Ravel, B.; Yacoby, Y.; Stern, E. A.; Dogan, F.; Rehr, J. J., The ferroelectric phase transition in $PbTiO_3$ from a local perspective. *Physica B: Condensed Matter* **1995,** *208-209*, 319-320.
14. Meyer, B.; Vanderbilt, D., Ab initio study of ferroelectric domain walls in $PbTiO_3$. *Physical Review B* **2002,** *65* (10), 104111.
15. Scott, J. F., Electrocaloric materials. *Annual Review of Materials Research* **2011,** *41* (1), 229-240.
16. *Physics of ferroelectrics: a modern perspective*. Springer: Berlin, Heidelberg, 2007.

17. Kumar, A.; Rabe, K. M.; Waghmare, U. V., Domain formation and dielectric response in $PbTiO_3$: A first-principles free-energy landscape analysis. *Physical Review B* **2013,** *87* (2), 024107.
18. Zhong, W.; Vanderbilt, D.; Rabe, K. M., Phase transitions in $BaTiO_3$ from first principles. *Physical Review Letters* **1994,** *73* (13), 1861-1864.
19. Waghmare, U. V.; Rabe, K. M., Ab initio statistical mechanics of the ferroelectric phase transition in $PbTiO_3$. *Physical Review B* **1997,** *55* (10), 6161-6173.
20. *Gibbs J.W. The collected works of J. Willard Gibbs: Vol. II Statistical Mechanics*. Yale University Press: New Haven, 1948.
21. Liu, Z.-K.; Wang, Y.; Shang, S.-L., Zentropy theory for positive and negative thermal expansion. *Journal of Phase Equilibria and Diffusion* **2022,** *43* (6), 598-605.
22. Liu, Z.-K., Theory of cross phenomena and their coefficients beyond Onsager theorem. *Materials Research Letters* **2022,** *10* (7), 393-439.
23. Liu, Z.-K., Thermodynamics and its prediction and CALPHAD modeling: Review, state of the art, and perspectives. *CALPHAD* **2023,** *82*, 102580.
24. Liu, Z.-K., Quantitative predictive theories through integrating quantum, statistical, equilibrium, and nonequilibrium thermodynamics. *Journal of Physics: Condensed Matter* **2024,** *36* (34), 343003.
25. Hohenberg, P.; Kohn, W., Inhomogeneous electron gas. *Physical Review* **1964,** *136* (3B), B864-B871.
26. Kohn, W.; Sham, L. J., Self-Consistent Equations Including Exchange and Correlation Effects. *Physical Review* **1965,** *140* (4A), A1133-A1138.
27. Sicron, N.; Ravel, B.; Yacoby, Y.; Stern, E. A.; Dogan, F.; Rehr, J. J., Nature of the ferroelectric phase transition in $PbTiO_3$. *Physical Review B* **1994,** *50* (18), 13168-13180.
28. Ravel, B.; Slcron, N.; Yacoby, Y.; Stern, E. A.; Dogan, F.; Rehr, J. J., Order-disorder behavior in the phase transition of $PbTiO_3$. *Ferroelectrics* **1995,** *164* (1), 265-277.
29. Fang, H. Z.; Wang, Y.; Shang, S. L.; Liu, Z. K., Nature of ferroelectric-paraelectric phase transition and origin of negative thermal expansion in $PbTiO_3$. *Physical Review B* **2015,** *91* (2), 024104.
30. Hew, N. L. E.; Shang, S.-L.; Liu, Z.-K., Predicting phase transitions in $PbTiO_3$ using zentropy through quasiharmonic phonon calculations. *Physical Review B* **2024,** *110* (18), 184103.
31. Neaton, J. B.; Ederer, C.; Waghmare, U. V.; Spaldin, N. A.; Rabe, K. M., First-principles study of spontaneous polarization in multiferroic $BiFeO_3$. *Physical Review B* **2005,** *71* (1), 014113.
32. Tan, H.; Takenaka, H.; Xu, C.; Duan, W.; Grinberg, I.; Rappe, A. M., First-principles studies of the local structure and relaxor behavior of $Pb(Mg_{1/3}Nb_{2/3})O_3$-$PbTiO_3$-derived ferroelectric perovskite solid solutions. *Physical Review B* **2018,** *97* (17), 174101.
33. Li, F.; Cabral, M. J.; Xu, B.; Cheng, Z.; Dickey, E. C.; LeBeau, J. M.; Wang, J.; Luo, J.; Taylor, S.; Hackenberger, W.; Bellaiche, L.; Xu, Z.; Chen, L.-Q.; Shrout, T. R.; Zhang, S., Giant piezoelectricity of Sm-doped $Pb(Mg_{1/3}Nb_{2/3})O_3$-$PbTiO_3$ single crystals. *Science* **2019,** *364* (6437), 264-268.
34. Otoničar, M.; Bradeško, A.; Fulanović, L.; Kos, T.; Uršič, H.; Benčan, A.; Cabral, M. J.; Henriques, A.; Jones, J. L.; Riemer, L.; Damjanovic, D.; Dražić, G.; Malič, B.; Rojac, T., Connecting the multiscale structure with macroscopic response of relaxor ferroelectrics. *Advanced Functional Materials* **2020,** *30* (52), 2006823.

35. Liu, H.; Shi, X.; Yao, Y.; Luo, H.; Li, Q.; Huang, H.; Qi, H.; Zhang, Y.; Ren, Y.; Kelly, S. D.; Roleder, K.; Neuefeind, J. C.; Chen, L.-Q.; Xing, X.; Chen, J., Emergence of high piezoelectricity from competing local polar order-disorder in relaxor ferroelectrics. *Nature Communications* **2023,** *14* (1), 1007.
